# Supplementary material for: Successful Preclinical Development of Gene Therapy for Recombinase-Activating Gene-1-Deficient SCID
Source: Mol Ther Methods Clin Dev. 2020 Mar 31;17:666–82. doi: 10.1016/j.omtm.2020.03.016 (PMC7163047; doi:10.1016/j.omtm.2020.03.016)
Supplement: Document S1. Supplemental Figures S1–S3 and Tables S1–S3 [file mmc1.pdf]

OMTM, Volume *17*

Supplemental Information

Successful Preclinical Development of Gene Therapy for Recombinase-Activating Gene-1-Deficient SCID

Laura Garcia-Perez, Marja van Eggermond, Lieke van Roon, Sandra A. Vloemans, Martijn Cordes, Axel Schambach, Michael Rothe, Dagmar Berghuis, Chantal Lagresle-Peyrou, Marina Cavazzana, Fang Zhang, Adrian J. Thrasher, Daniela Salvatori, Pauline Meij, Anna Villa, Jacques J.M. Van Dongen, Jaap-Jan Zwaginga, Mirjam van der Burg, H. Bobby Gaspar, Arjan Lankester, Frank J.T. Staal, and Karin Pike-Overzet

Suppl. Figure 1: Optimal SIN LV choice

A)

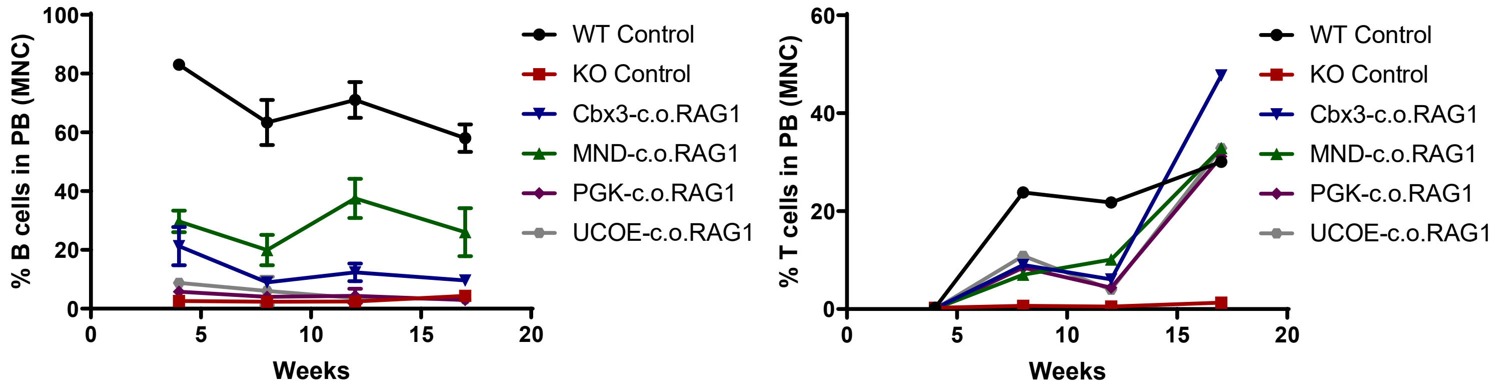

B)

In vitro	Cbx3-MND	MND	PGK	UCOE
Physical titer	++	++	+	+/-
Functional titer	+	++	+	+/-
Transgene expression	+	+	-	-
Promoter strength	++	+	-	+

C)

In vivo	Cbx3-MND	MND
B cell development BM	-	+
Thymic reconstitution	-	+
Mature immune cells	-	+
Immunoglobulins	-	+
T cell repertoire	+/-	+/-
Safety (IVIM)	+/-	+/-
Omenn-like syndrome in mice (due to low c.o.RAG1 expression)	Observed	Not observed

Suppl. Figure 2: Immune development after gene therapy in Rag1-/- mouse model

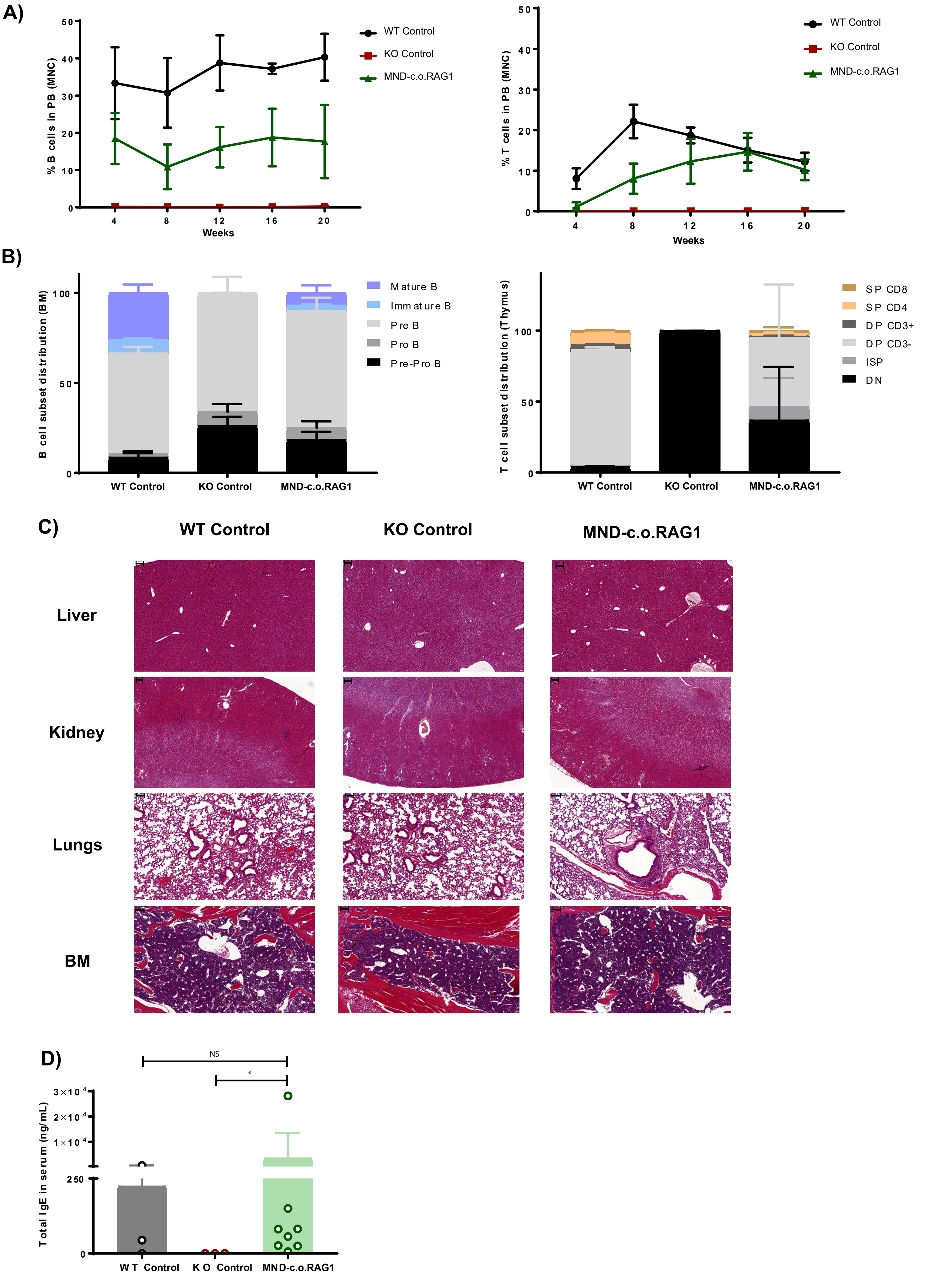

Suppl. Figure 3 **Human immune reconstitution after CD34+ MND-c.o.RAG1 transplantation**

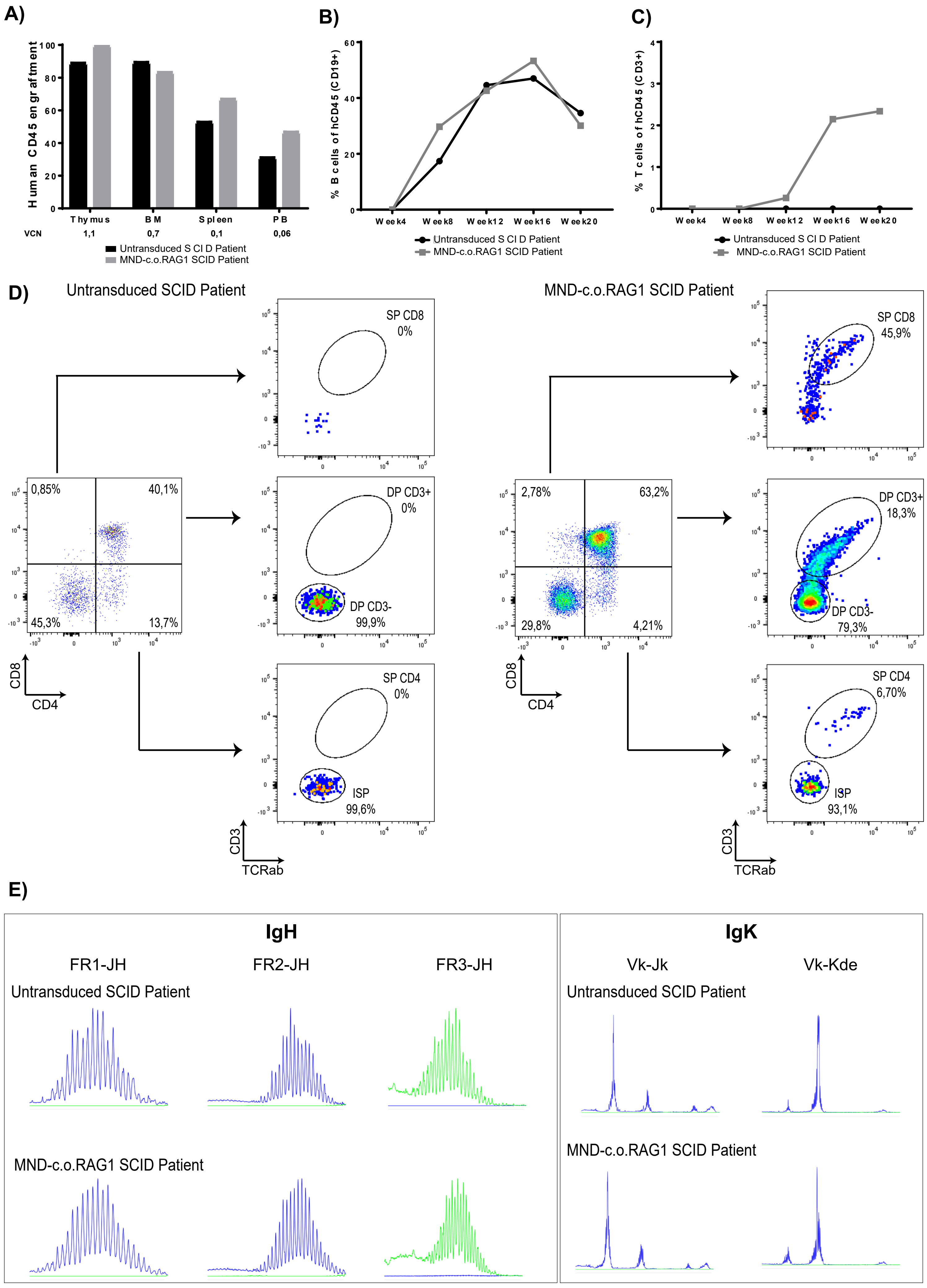

Supplemental Figure:

Figure S1: Choice of the optimal SIN Lentiviral plasmid

A) Percentage of B cells (CD11b/CD43$^-$B220$^+$ cells; left panel) and T cells (CD3$^+$TCRαβ$^+$ cells; right panel) over time in PB after stem cell transplantation with the different constructs (Cbx3.MND-c.o.RAG1, MND-c.o.RAG1, PGK-c.o.RAG1 and UCOE-c.o.RAG1) (Data from 2 independent experiments, total of 6 or 7 mice/group). **B)** Summary of the *in vitro* decision criteria (from 3 independent lentiviral batches) taken into account for the choice of the most optimal plasmid to correct RAG1 deficiency. **C)** Summary of the *in vivo* decision criteria (2 independent experiments with total of 6 or 7 mice per group) taken into account for the choice of the most optimal plasmid to correct RAG1 deficiency.

Figure S2: Immune development after MND-c.o.RAG1 gene therapy in Rag1$^{-/-}$ mouse model

A) Percentage of B cells (CD11b/CD43$^-$B220$^+$ cells; left panel) and T cells (CD3$^+$TCRαβ$^+$ cells; right panel) over time in PB after stem cell transplantation with the clinical MND-c.o.RAG1 batch (3 WT control mice, 3 KO control mice and 8 MND-c.o.RAG1 mice). **B)** B-cell development subsets distribution in BM (left panel) and T-cell development populations distribution in the thymus (right panel) 24 weeks after SC transplantation. Graphs represent the means and standard deviation of 3 mice for control groups and 8 mice in the gene therapy group. **C)** Histologic analysis of the liver (scale bar = 100µm), kidney (scale bar=200µm), lungs (scale bar=100µm) and BM (scale=100µm) stained with hematoxylin and eosin. Representative images from WT Control, KO Control and MND-c.o.RAG1 mice. **D)** Quantification of total IgE in serum by ELISA. Each dot represents a value obtained in one mouse (3 mice/control group, 8 MND-c.o.RAG1 mice). Mann-Whitney test (two tailed, *p<0,05; **p<0,01)

Figure S3: Human immune reconstitution after CD34+ MND-c.o.RAG1 transplantation

A) Percentage of human chimerism (hCD45$^+$/(hCD45$^+$mCD45$^+$) in immune organs of NSG mice transplanted with CD34$^+$ SCID patient cells and CD34$^+$ SCID patient cells transduced with MND-c.o.RAG1, 24 weeks after transplantation (1 NSG mouse per condition). **B)** Human B-cell percentage (CD19$^+$ cells per total hCD45$^+$ cells) over time in peripheral blood during transplantation. **C)** Human T-cell development (CD3$^+$ cells per total hCD45$^+$ cells) over time in PB during transplantation. **D)** Flow cytometry analysis of thymocytes 24 weeks after transplantation showing T-cell development through the different stages. **E)** Human IgH and IgK repertoire analysis of isolated DNA from NSG BM (SCID patient and SCID MND-c.o.RAG1) using IgH + IgK B-Cell Clonality Assay. (x-axis indicates fragment sizes; y-axis shows the fluorescence intensity of the runoff products).

Table S1
Organs list

Organs	Immune phenotyping	Pathology	Vector Biodistribution
Adrenal gland		x	
Brain		x	x
Cecum		x	
Colon		x	
Duodenum		x	
Gastrocnemius		x	x
Gonalds		x	x
Head-Eyes		x	
Heart		x	x
Ileum		x	
Jejunum		x	x
Kidney		x	x
Limb (front and back)	x	x	x
Liver		x	x
Lung		x	x
Lymph Node (iliac)			x
Lymph Node (Lumbar)		x	
Lymph Node (mesenteric)		x	
Lymph Node (sacral)		x	
Lymph Node (submand.)		x	
Pancreas		x	x
Rectum		x	
Skin		x	
Spinal Cord		x	
Spleen	x	x	x
Sternum		x	
Stomach		x	x
Thymus	x	x	x
Urinary Bladder		x	x

Table S2: Antibody list

Anti-mouse Antibody	Fluorochrome	Clone	Company	Identifier	
				Catalog number	RRID
CD3e	Biotin	145-2C11	BD Bioscience	553060	AB_394593
CD4	PE-Cy7	RM4-5	eBioscience	25-0042-82	AB_469578
CD8a	PerCP	53-6.7	BioLegend	100732	AB_893423
CD11b	Biotin	M1/70	Biolegend	101204	AB_312787
CD19	APC	1D3	BD Bioscience	550992	AB_398483
CD23	Pe-Cy7	B3B4	eBioscience	25-0232-81	AB_469603
CD43	Biotin	S7	BD Bioscience	553269	AB_2255226
CD43	PE	S7	BD Bioscience	553271	AB_394748
CD44	APC-Cy7	IM7	BD Bioscience	560568	AB_1727481
CD45	FITC	30-F11	BD Bioscience	553079	AB_394609
CD45R/B220	PerCP	RA3-6B2	Biolegend	103233	AB_893355
CD45R/B220	Pe-Cy7	RA3-6B2	eBioscience	25-0452	AB_2341160
CD62L	APC	MEL-14	Biolegend	104411	AB_313098
CD93	APC	AA4.1	eBioscience	17-5892	AB_469466
CD138	PE	281-2	BD Bioscience	553714	AB_395000
IgD	ef450	11-26c	eBioscience	48-5993-80	AB_1272239
IgM	FITC	II/41	BD Bioscience	553437	AB_394857
TCRβ	FITC	H57-597	BD Bioscience	553171	AB_394683
TCRγδ	PE	GL3	BD Bioscience	553178	AB_394689
Streptavidin	APC-Cy7	-	BD Bioscience	554063	AB_10054651
Streptavidin	ef450	-	eBioscience	48-4317-82	AB_10359737

Anti-human Antibody	Fluorochrome	Clone	Company	Identifier	
				Catalog number	RRID
CD1a	APC	HI149	BD Bioscience	559775	AB_398669
CD3	FITC	SK7	BD Bioscience	340542	AB_400051
CD3	BV786	SK7	BD Bioscience	563800	AB_2744384
CD4	PE	SK3	BD Bioscience	345769	AB_2728699
CD4	APC-Cy7	RPA-T4	BD Bioscience	557871	AB_396913
CD5	FITC	UCHT2	BD Bioscience	561896	AB_10894588
CD7	Pe-Cy5	M-T701	BD Bioscience	555362	AB_395765
CD8	Pe-Cy7	SK1	BD Bioscience	335805	AB_399980
CD8	BV650	RPA-T8	Biolegend	301041	AB_11125174
CD10	Biotin	SN5c	eBioscience	13-0108-82	AB_763433
CD13	APC	WM15	BD Bioscience	557454	AB_398624
CD16	PE	B73.1	BD Bioscience	347617	AB_400331
CD19	Pe-Cy7	HIB19	eBioscience	25-0199-41	AB_1582279
CD19	APC-Cy7	HIB19	BD Bioscience	557791	AB_396873
CD20	PE	L27	BD Bioscience	346581	AB_400251
CD33	APC	P67.6	BD Bioscience	551378	AB_398502
CD34	PE	8G12	BD Bioscience	348057	AB_400371
CD34	PE-CF594	581	BD Bioscience	562383	AB_11154586
CD34	APC	581	BD Bioscience	555824	AB_398614
CD38	Pe-Cy7	HIT2	eBioscience	25-0389-42	AB_1724057
CD45	V450	HI30	BD Bioscience	560367	AB_1645573

CD45RA	FITC	L48	BD Bioscience	347723	AB_400343
CD45RA	BV510	HI100	BD Bioscience	563031	AB_2722499
CD49f	PerCP-ef710	ebioGoH3	eBioscience	46-0495-82	AB_10670075
CD56	PE	MY31	BD Bioscience	340685	AB_400088
CD56	APC-Cy7	HCD56	Biolegend	318332	AB_10896424
CD62L	BV605	DREG-56	Biolegend	304833	AB_2562129
CD90	APC	5E10	eBioscience	17-0909-42	AB_11042579
IgD	Biotin	IA6-2	BD Bioscience	555777	AB_396112
IgM	PerCP-Cy5.5	MHM-88	Biolegend	314512	AB_2076098
TCRαβ	PerCP-Cy5.5	IP26	Biolegend	306723	AB_2563001
TCRγδ	APC	B1	BD Bioscience	555718	AB_398611
Streptavidin	PerCP-Cy5.5	-	BD Bioscience	551419	
Streptavidin	Pe-Cy7	-	BD Bioscience	557598	AB_10049577

**Tissue staining Antibody**	**Fluorochrome**	**Clone**	**Company**	**Identifier**	
				**Catalog number**	**RRID**
FOXP3 Antibody , ABfinity™ Rabbit Monoclonal	-	5H10L18	Thermofisher	700914	-
Keratine 5/6 Monoclonal	-	D5/16 B4	Dako	GA780	-

**ELISA Antibody**	**Fluorochrome**	**Clone**	**Company**	**Identifier**	
				**Catalog number**	**RRID**
Anti-mouse IgG	Unlabeled	-	SouthernBiotech	Cat# 1030-01	-
Anti-mouse IgM	Unlabeled	-	SouthernBiotech	Cat# 1020-01	-
Anti-mouse IgE	Unlabeled	-	SouthernBiotech	Cat# 1110-01	-
Anti-mouse IgG	Biotin-conjugated	-	SouthernBiotech	Cat# 1030-08	-
Anti-mouse IgM	Biotin-conjugated	-	SouthernBiotech	Cat# 0101-08	-
Anti-mouse IgE	Biotin-conjugated	-	SouthernBiotech	Cat# 1002-08	-
Anti-human IgM	Unlabeled	-	Jackson Immuno Research Laboratories	Cat# 109-005-129	-
Anti-human IgM	Biotin-conjugated	-	Novex Life Technologies	Cat# A24486	-
Streptavidin HRP		-	Jackson Immuno Research Laboratories	Cat# 016-030-084	-

Table S3
List of primers and probes

Determination VCN and c.o.Rag1 expression

Description	Orientation	DNA sequence 5'-3'
ABL1	FW	5'-TGGAGATAACACTCTAAGCATAACTAAAGGT-3'
	RV	5'-GATGTAGTTGCTTGGGACCCA-3'
	Probe	5'FAM-CCATTTTTGGTTTGGGCTTCACACCATT- TAMRA 3'
c.o.Rag1	FW	5' CAACTGCAAGCACGTGTTCTG 3'
	RV	5' GCAGTAGCTGCCCATCACTTT 3'
	Probe	5'FAM AGAGTGTGCATCCTGCGGTGCCT TAMRA 3'
PTBP2	FW	5'-TCTCCATTCCCTATGTTCATGC-3'
	RV	5'-GTTCCCGCAGAATGGTGAGGTG-3'
	Probe	[JOE]-ATGTTCCTCGGACCAACTTG-[BHQ1]
WPRE	FW	5'- GAGGAGTTGTGGCCCGTTGT-3'
	RV	5'-TGACAGGTGGTGGCAATGCC-3'
	Probe	[6FAM]-CTGTGTTTGCTGACGCAAC-[BHQ1]

Repertoire Analysis (murine)

Description	Orientation	DNA sequence
***V* gene segment-specific oligonucleotide**		**(5' -> 3', coding strand)**
mVβ1	FW	CTGAATGCCCAGACAGCTCCAAGC
mVβ2	FW	TCACTGATACGGAGCTGAGGC
mVβ3.1	FW	CCTTGCAGCCTAGAAATTCAGT
mVβ4	FW	GCCTCAAGTCGCTTCCAACCTC
mVβ5.1	FW	CATTATGATAAAATGGAGAGAGAT
mVβ5.2	FW	AAGGTGGAGAGAGACAAAGGATTC
mVβ5.3[#]	FW	AGAAAGGAAACCTGCCTGGTT
mVβ6	FW	CTCTCACTGTGACATCTGCCC
mVβ7	FW	TACAGGGTCTCACGGAAGAAGC
mVβ8.1	FW	CATTACTCATATGTCGCTGAC
mVβ8.2	FW	CATTATTCATATGGTGCTGGC
mVβ8.3	FW	TGCTGGCAACCTTCGAATAGGA
mVβ9	FW	TCTCTCTACATTGGCTCTGCAGGC
mVβ10	FW	ATCAAGTCTGTAGAGCCGGAGGA
mVβ11	FW	GCACTCAACTCTGAAGATCCAGAGC
mVβ12	FW	GATGGTGGGGCTTTCAAGGATC
mVβ13	FW	AGGCCTAAAGGAACTAACTCCCAC
mVβ14	FW	ACGACCAATTCATCCTAAGCAC
mVβ15	FW	CCCATCAGTCATCCCAACTTATCC
mVβ16	FW	CACTCTGAAAATCCAACCCAC
mVβ17[#]	FW	AGTGTTCCTCGAACTCACAG
mVβ18	FW	CAGCCGGCCAAACCTAACATTCTC
mVβ19[#]	FW	CTGCTAAGAAACCATGTACCA
mVβ20	FW	TCTGCAGCCTGGGAATCAGAA
**C gene segment specific oligonucleotide**		**(5' -> 3', non-coding strand)**
muTCB1-FAM	RV	FAM-CTTGGGTGGAGTCACATTTCTC
