# Supplementary material for: Successful Preclinical Development of Gene Therapy for Recombinase-Activating Gene-1-Deficient SCID
Source: Mol Ther Methods Clin Dev. 2020 Mar 31;17:666–82. doi: 10.1016/j.omtm.2020.03.016 (PMC7163047; doi:10.1016/j.omtm.2020.03.016)
Supplement: Document S2. Document plus Supplemental Information [file mmc2.pdf]

Molecular Therapy
Methods & Clinical Development
Original Article

AMERICAN SOCIETY of GENE & CELL THERAPY

Successful Preclinical Development of Gene Therapy for Recombinase-Activating Gene-1-Deficient SCID

Laura Garcia-Perez,[1] Marja van Eggermond,[1] Lieke van Roon,[1] Sandra A. Vloemans,[1] Martijn Cordes,[1] Axel Schambach,[2] Michael Rothe,[2] Dagmar Berghuis,[3] Chantal Lagresle-Peyrou,[4,5,6] Marina Cavazzana,[4,5,6] Fang Zhang,[7] Adrian J. Thrasher,[7] Daniela Salvatori,[8,9,10,11] Pauline Meij,[9] Anna Villa,[10] Jacques J.M. Van Dongen,[1] Jaap-Jan Zwaginga,[1] Mirjam van der Burg,[3] H. Bobby Gaspar,[7] Arjan Lankester,[3] Frank J.T. Staal,[1] and Karin Pike-Overzet[1]

[1]Department of Immunohematology and Blood Transfusion, Leiden University Medical Center, 2333ZA Leiden, the Netherlands; [2]Institute of Experimental Hematology, Hannover Medical School, 30625 Hannover, Germany; [3]Willem-Alexander Children's Hospital Department of Pediatrics, Leiden University Medical Center, 2333ZA Leiden, the Netherlands; [4]Biotherapy Clinical Investigation Center, Groupe Hospitalier Universitaire Ouest, Assistance Publique-Hôpitaux de Paris, INSERM CIC 1416, Paris, France; [5]Laboratory of Human Lymphohematopoiesis, INSERM UMR 1163, Imagine Institute and Paris Descartes University-Sorbonne Paris Cité, 75015 Paris, France; [6]Department of Biotherapy, Necker Children's Hospital, Assistance Publique-Hôpitaux de Paris, 75015 Paris, France; [7]Molecular and Cellular Immunology, Great Ormond Street Institute of Child Health, and Great Ormond Street Hospital NHS Trust, London WC1N 1EH, UK; [8]Central Laboratory Animal Facility, Pathology Unit, Leiden University Medical Center, 2333ZA Leiden, the Netherlands; [9]Department of Pharmacy, Leiden University Medical Center, 2333ZA Leiden, the Netherlands; [10]Pathogenesis and Treatment of Immune and Bone Diseases Unit, San Raffaele Telethon Institute for Gene Therapy (SR-Tiget), IRCCS San Raffaele Scientific Institute, 20132 Milan, Italy; [11]Anatomy and Physiology Division, Clinical Sciences, Faculty of Veterinary Medicine, Utrecht University, Yalelaan1, 3584CL Utrecht, the Netherlands

Recombinase-activating gene-1 (RAG1)-deficient severe combined immunodeficiency (SCID) patients lack B and T lymphocytes due to the inability to rearrange immunoglobulin and T cell receptor genes. Gene therapy is an alternative for those RAG1-SCID patients who lack a suitable bone marrow donor. We designed lentiviral vectors with different internal promoters driving codon-optimized *RAG1* to ensure optimal expression. We used *Rag1*$^{-/-}$ mice as a preclinical model for RAG1-SCID to assess the efficacy of the various vectors. We observed that B and T cell reconstitution directly correlated with *RAG1* expression. Mice with low *RAG1* expression showed poor immune reconstitution; however, higher expression resulted in phenotypic and functional lymphocyte reconstitution comparable to mice receiving wild-type stem cells. No signs of genotoxicity were found. Additionally, RAG1-SCID patient CD34^{+} cells transduced with our clinical RAG1 vector and transplanted into NSG mice led to improved human B and T cell development. Considering this efficacy outcome, together with favorable safety data, these results substantiate the need for a clinical trial for RAG1-SCID.

INTRODUCTION

Severe combined immunodeficiency (SCID) is a life-threatening disorder of the adaptive immune system.[1] In all forms of SCID, the development of T cells in the thymus is arrested due to genetic defects in genes essential for this complex process, while concomitant deficiencies in B lymphocytes and natural killer (NK) cells depend on the SCID genotype. Affected infants are born with a severe T lymphocyte deficiency and die within the first year of life unless effective treatment is given. Curative treatment options are limited and confined to allogeneic hematopoietic stem cell transplantation (HSCT)[2,3] and autologous stem cell gene therapy (GT).[4,5]

More than 20 different genes have been shown to be causative for SCID.[1] Three major types of SCID exist, which include the common γ-chain cytokine deficiencies, mainly due to defects in the IL2Rγ chain (which is also termed the common γ-chain). Deficiencies in JAK3 and IL7Ra are much more rare but also fall into this category. The second type of SCID concerns metabolic enzymes that affect highly proliferating cells such as immature thymocytes. Adenosine deaminase (ADA) deficiency is the prototype disease for this subtype, but other deficiencies have also been found, for instance purine nucleoside phosphorylase (PNP) deficiency. The third major type of SCID is formed by recombination deficiencies. In these types of SCID the recombination machinery that is responsible for variable diversity joining (V(D)J) recombination of T cell receptor (TCR) and immunoglobulin (Ig) genes is affected. Examples are recombination-activating gene-1 (RAG1), RAG2 deficiency, and Artemis mutations. The exact

Received 13 March 2020; accepted 23 March 2020;
https://doi.org/10.1016/j.omtm.2020.03.016.

Correspondence: Frank J.T. Staal, Department of Immunohematology and Blood Transfusion, Leiden University Medical Center, 2333ZA Leiden, the Netherlands.
E-mail: f.j.t.staal@lumc.nl

 © 2020 The Authors.
This is an open access article under the CC BY license (http://creativecommons.org/licenses/by/4.0/).

nature of the T cell developmental arrests in SCID patients has been difficult to elucidate because thymic biopsies cannot be taken; however, recent functional experiments using bone marrow (BM) stem/progenitor cells from SCID patients has shown that most mutations lead to very early blocks in thymic differentiation.[6–8]

During the last 15 years, clinical trials of gene therapy for two major forms of SCID (SCID-X1 and ADA SCID) have shown significant safety and efficacy in correcting the immunodeficiency and allowing children to live normal functional lives.[4,5,9–15] This despite the occurrence of T cell acute lymphoblastic leukemia (T-ALL) as a severe adverse effect in some of these early trials,[16–19] which has led to an impetus to further develop safer vectors, the so-called self-inactivating (SIN) vectors.[20–22]

For the recombination deficiencies, major steps have been made for correcting RAG1, RAG2, and Artemis deficiency. Artemis gene therapy is closest to clinical implementation, and a first clinical trial has started in the US.[23] For RAG1-SCID, several attempts to develop gene therapy have been made in the past, first with the now no longer acceptable γ-retroviral vectors,[24] later with SIN lentiviral vectors.[25,26] Our previous work reported successful restoration of Rag1 deficiency using SIN lentiviral vector technology and codon-optimized *RAG1* (*c.o.RAG1*);[26] however, with a lentiviral (LV) vector backbone that is not suitable for large-scale guanosine monophosphate (GMP) production and with a promoter that may lead to genotoxicity (see below). In this previous report, we obtained full restoration of peripheral T cell numbers after 5 months using spleen focus-forming virus (SFFV), approximately 35% of normal B cell numbers, and, importantly, a polyclonal TCR and B cell receptor (BCR) repertoire and full restoration of serum Ig levels, allowing functional responses after immunization with the T cell-dependent antigens.

However, others have argued that by using this approach, it is not possible to fully correct the RAG1 immune deficiency,[27] and that oligoclonal T cells could develop, reminiscent of human Omenn syndrome, a disorder known to arise from hypomorphic RAG mutations, resulting in low recombinase activity. We have stated elsewhere[28] that these discrepant results can likely be explained by differences in the expression levels and low transduction efficiencies obtained for the therapeutic gene, *RAG1*. Herein we report that successful restoration of the RAG1 deficiency can be obtained using SIN LV vectors that are clinically acceptable and, importantly, at low vector copy numbers (i.e., ~1 copy per cell).

A disadvantage of our previous LVs was the use of the so-called RRL backbone, which gives relatively low titers in scaled-up virus productions needed for clinical application. Therefore, we switched to the CCL backbone that has been widely used clinically. In addition, the SFFV promoter sequence that was the most successful in our hands has become less attractive due to the assumed high risk of insertional mutagenesis.[29] Therefore, we set out to develop a new set of SIN lentiviral vectors to express *c.o.RAG1* with different types of promoters and to test whether they could correct Rag1 deficiency in a preclinical mouse model with low vector copy numbers, as to carry a lower risk of insertional mutagenesis. Through serendipitous effects in the viral production and titration of viral transduction, we obtained a whole range of *RAG1* expression *in vivo* ranging from very low to close to wild-type (WT) levels. This allowed us to directly address the effects of differences in *RAG1* expression in a gene therapy setting. In addition, it has enabled us to choose a new SIN LV vector that functionally corrects the Rag1 deficiency *in vivo* in mice. The MND-c.o.RAG1 is now the vector of choice capable of high *RAG1* expression that is produced at clinical grade for an international multi-center RAG1-SCID gene therapy trial that is planned in the near future.

RESULTS

MND Promoter as the Optimal Vector to Correct Rag1 Deficiency

At the onset of this project, we constructed four different SIN LV transfer plasmids in the CCL backbone and tested four different promoters: PGK (human phosphoglycerate kinase [PGK]-1 promoter, nucleotides 5–516; GenBank: M11958)[30]; MND (myeloproliferative sarcoma virus enhancer, negative control region deleted, dl587rev primer binding site substituted promoter)[31]; UCOE (the modified chromatin-remodeling element, devoid of unwanted splicing activity and minimized read-through activity[32]; and a tandem combination of UCOE and MND (Cbx3.MND), which was used to drive expression of a codon-optimized version of *RAG1* (Figure 1A).

Recombinant lentiviruses were produced at small and large scales to evaluate virus production and *in vitro* expression efficiency of the different vectors. The transfer vectors in conjunction with GAG-Pol, REV, and vesicular stromatitis virus G protein (VSV-G) plasmids were transiently transfected into 293T cells to produce the different lentiviruses. The number of infectious particles of the small and large virus batches was assessed before and after concentration by qPCR. Consistently, both the small and large batches of UCOE-c.o.RAG1 lentivirus (both unconcentrated and concentrated) had a significantly lower number of infectious genomes per milliliter compared to the other vectors (Figures 1B and 1C), highlighting a difficulty to scale up its production. These lentiviruses were subsequently used to transduce lineage-negative BM cells from Rag1-deficient mice in order to determine their functional characteristics under conditions relevant for *in vivo* application. We found that UCOE-c.o.RAG1 reached a lower viral copy number (VCN) (Figure 1D) than did the other vectors, and PGK-c.o.RAG1 was the vector with the lowest promoter strength (Figure 1F). Unfortunately, both PGK and UCOE-c.o.RAG1 only resulted in low levels of *c.o.RAG1* expression (Figure 1E) whereas quite high levels are known to be required for immune reconstitution.[24,26,33] Indeed, an *in vivo* pilot experiment where Rag1-deficient mice were transplanted with WT stem cells, mock-transduced Rag1-deficient stem cells, or gene therapy-treated stem cells using the four different promoters revealed that the promoter strength and essentially the level of *c.o.RAG1* are crucial to obtain adequate immune reconstitution (two independent pilot experiments, total of six or seven mice per group). Immune reconstitution of these mice was followed in the peripheral blood (PB) every 4 weeks, showing that

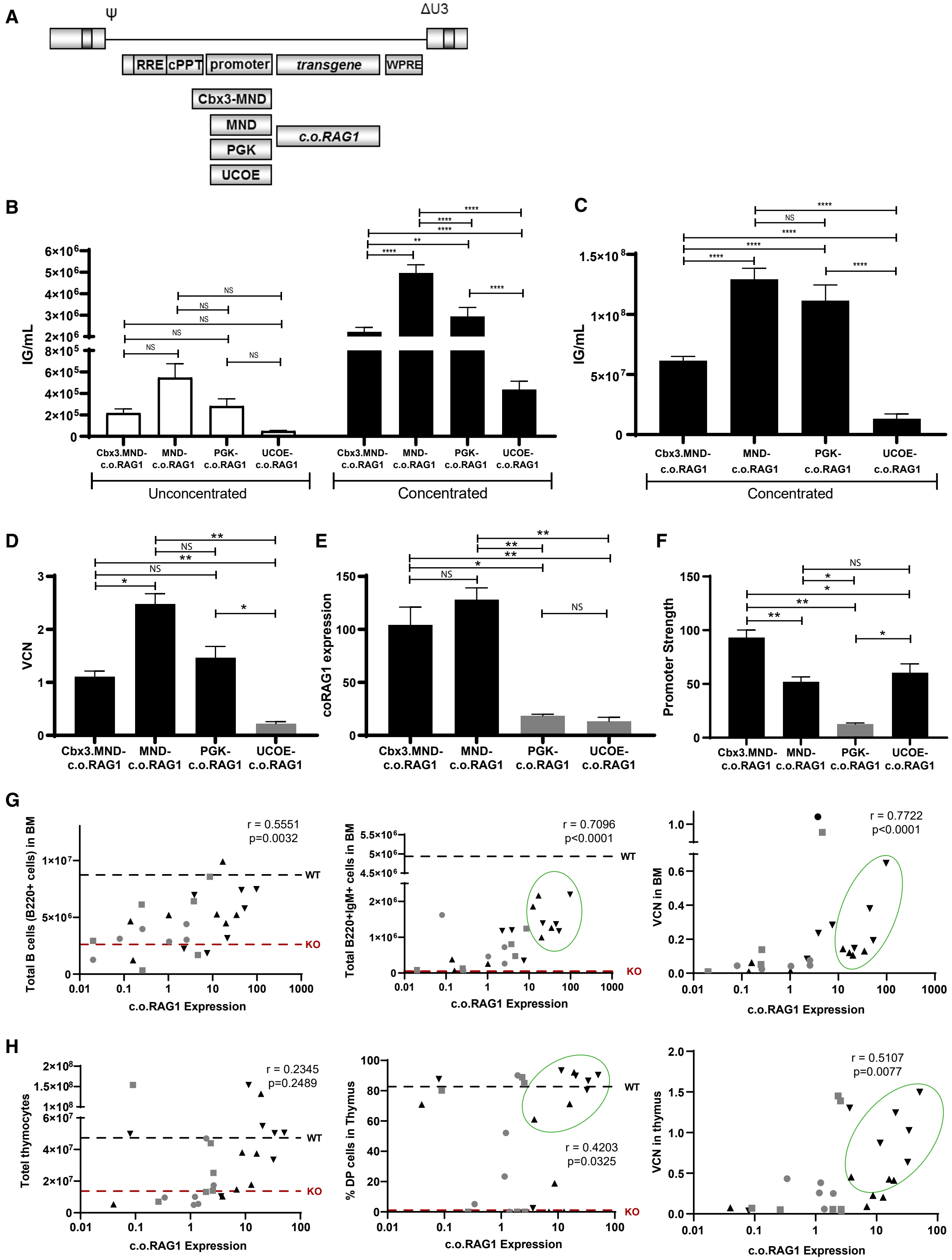

(legend on next page)

reconstitution of B cells and T cells was achieved in the different gene therapy group to different extents (Figure S1A). Reflecting the known promoter strengths of these four vectors, a wide range of c.o.*RAG1* expression was created by this initial experiment. Interestingly, 16 weeks after transplantation, we observed a clear linear correlation between the expression of *c.o.RAG1* achieved in the BM and the number of B cells ($B220^{+}IgM^{+}$ cells) generated (Figure 1G, left and middle panels). For T cells, we observed that there was a threshold of minimal c.o.*RAG1* expression to develop an active double-positive (DP) CD4 and CD8 population in the thymus, roughly at 10-fold the housekeeping control level (Figure 1H, left and middle panels). Mice reconstituted with stem cells having lower *c.o.RAG1* expression than this threshold barely reconstituted thymic T cell development. Accordingly, B and T cell reconstitution was consistently achieved in the BM and in the thymus when *c.o.RAG1* expression at 10-fold the housekeeping control level or higher could be achieved. This expression level was mainly reached with VCNs of 1 and lower (Figures 1G and 1H, right panels) using the high-expressing vectors such as Cbx3.MND and MND-c.o.RAG1 (black symbols in Figures 1G and 1H). We considered that mice achieved immune reconstitution when B and T cell development was successful (overcoming the early developmental block) and the cells were functional, with a diverse TCR Vβ repertoire and without signs of toxicity or adverse side effects (Figures 1G and 1H, green circle). In the low *c.o.RAG1* expression mice (gray dots, mainly PGK and UCOE promoter), we found a number of mice (n = 4 out of 9) that developed skin rashes and wasting during the course of the experiments, which resulted in the death of some mice (similar to the features due to low RAG1 activity described previously[27]), whereas the animals in the higher *c.o.RAG1* expression group (black dots, Cbx3.MND and MND promoter) as well as the animals that received WT cells or uncorrected Rag1 knockout (KO) cells did not display any health problems. Collectively, our *in vitro* and *in vivo* pilot data highlight the importance of achieving sufficient *c.o.RAG1* expression, at VCN around or below 1, in order to obtain successful immune reconstitution, which was only accomplished using Cbx3.MND-c.o.RAG1 and MND-c.o.RAG1 lentiviruses (Figure S1B).

To better compare both vectors, an additional *in vivo* reconstitution experiment was done, with more comparable VCNs. Rag1-deficient mice transplanted with WT stem cells, mock-transduced Rag1 KO stem cells, Cbx3.MND-c.o.RAG1-treated stem cells (starting VCN of 0.95), or MND-c.o.RAG1-treated stem cells (starting VCN of 1.1) were extensively analyzed 16 weeks after transplantation by flow cytometry and qPCR for VCN measuring WPRE (woodchuck hepatitis virus posttranscriptional regulatory element) and expression of the therapeutic gene c.o.*RAG1*. Mice were sacrificed after 4 months, and immune organs were analyzed by flow cytometry (pilot experiment with a total of three mice per group). Restoration of $IgM^{+}B220^{+}$ B cells (Figure 2A) in the BM was seen in mice treated with WT stem cells and MND-c.o.RAG1-treated gene therapy mice and occasionally in mice with Cbx3.MND elements, even with a comparable VCN. Mock-transduced Rag1 KO stem cells did not restore B cell development, where cells were blocked at the precursor (pre-)B cell stage, as expected. In contrast, in gene-therapy-treated mice the arrest in B cell development was alleviated and immature and mature B cells developed (Figure 2B, left panel). MND-c.o.RAG1 gene therapy mice successfully developed all B cell developmental subsets in the BM, similarly to WT transplanted mice and significantly different from the mock KO transplanted mice. We observed that even though B cell development in BM was satisfactory, B cell numbers detected in the PB were significantly lower than in the WT situation (Figure 2B, right panel). However, B cell functionality was fully restored to WT degree as the levels of IgG and IgM detected in serum were comparable to WT transplanted mice (Figure 2G). We next analyzed the thymus for T cell marker expression using (among other markers) CD4 and CD8. Proper T cell development with a full spectrum of DP and single-positive (SP) CD4 or CD8 developmental stages was observed with WT and MND-c.o.RAG1 cells, but not with Cbx3.MND-c.o.RAG1 cells, where mice showed an exhausted thymus phenotype with mature CD4 and CD8 SP cells but not DP cells at

Figure 1. Selecting the Optimal SIN LV Plasmid: Virus Production And *In* Vitro Efficiency

(A) Four different SIN LV plasmids in the CCL backbone carrying different promoters (Cbx3.MND, MND, PGK, and UCOE promoter) were tested to drive expression of a codon-optimized version of *RAG1*. (B) Production of lentivirus batches with the different constructs. The number of infective particles (infectious genomes/mL) from unconcentrated and concentrated small batches was determined. Three independent lentivirus small batches per plasmid were produced and analyzed (two-way ANOVA test; *p < 0.05, **p < 0.01). (C) Production of lentivirus batches on a large scale with the different constructs. The number of infectious genomes/mL after concentration of large lentiviruses batches was determined. (D) Transduction efficiency of the different SIN lentiviruses in murine lineage-negative cells. VCN was determined by WPRE determination on genomic DNA. Three independent lentivirus batches per plasmid were produced and analyzed (one-way ANOVA test; *p < 0.05, **p < 0.01. (E) Determination of transgene expression in the transduced cells by the different constructs. *c.o.RAG1* expression relative to *ABL1* was determined by qPCR. Three independent lentivirus batches per plasmid were produced and analyzed (one-way ANOVA test; *p < 0.05, **p < 0.01. (F) Determination of the promoter strength (*c.o.RAG1* expression/VCN) of the different plasmids. Three independent lentivirus batches per plasmid were produced and analyzed (one-way ANOVA test; *p < 0.05, **p < 0.01). (G) Total number of $B220^{+}$ cells (left panel) and total number of $B220^{+}IgM^{+}$ cells (middle panel) correlated with the expression of *c.o.RAG1* in BM. The correlation between VCN and c.o.RAG1 expression in BM of immune reconstituted mice is shown (right panel) (▲, Cbx3.MND; ▼, MND; ■, PGK; ●, UCOE promoters; gray indicates low-expressing plasmids; black indicates high0expressing plasmids; green circles indicate mice with acceptable immune B and T cell reconstitution). Data shown represent two independent *in vivo* experiments with in total six or seven mice per group. Each dot represents one mouse. Nonparametric Spearman r correlation, two-tailed; **p < 0.01, ***p < 0.001, ****p < 0.0001. (H) Correlation between total thymocytes (left panel) and DP cells (middle panel) with *c.o.RAG1* expression in the thymus. Correlation between VCN and *c.o.RAG1* expression in the thymus of immune reconstituted mice (right panel) (▲, Cbx3.MND; ▼, MND; ■, PGK; ●, UCOE promoters; gray indicates low-expressing plasmids; black indicates high-expressing plasmids; green circles indicate mice with acceptable immune B and T cell reconstitution). Data shown represent two independent *in vivo* experiments with in total six or seven mice per group. Each dot represents one mouse. Nonparametric Spearman r correlation, two-tailed; **p < 0.01, ***p < 0.001, ****p < 0.0001.

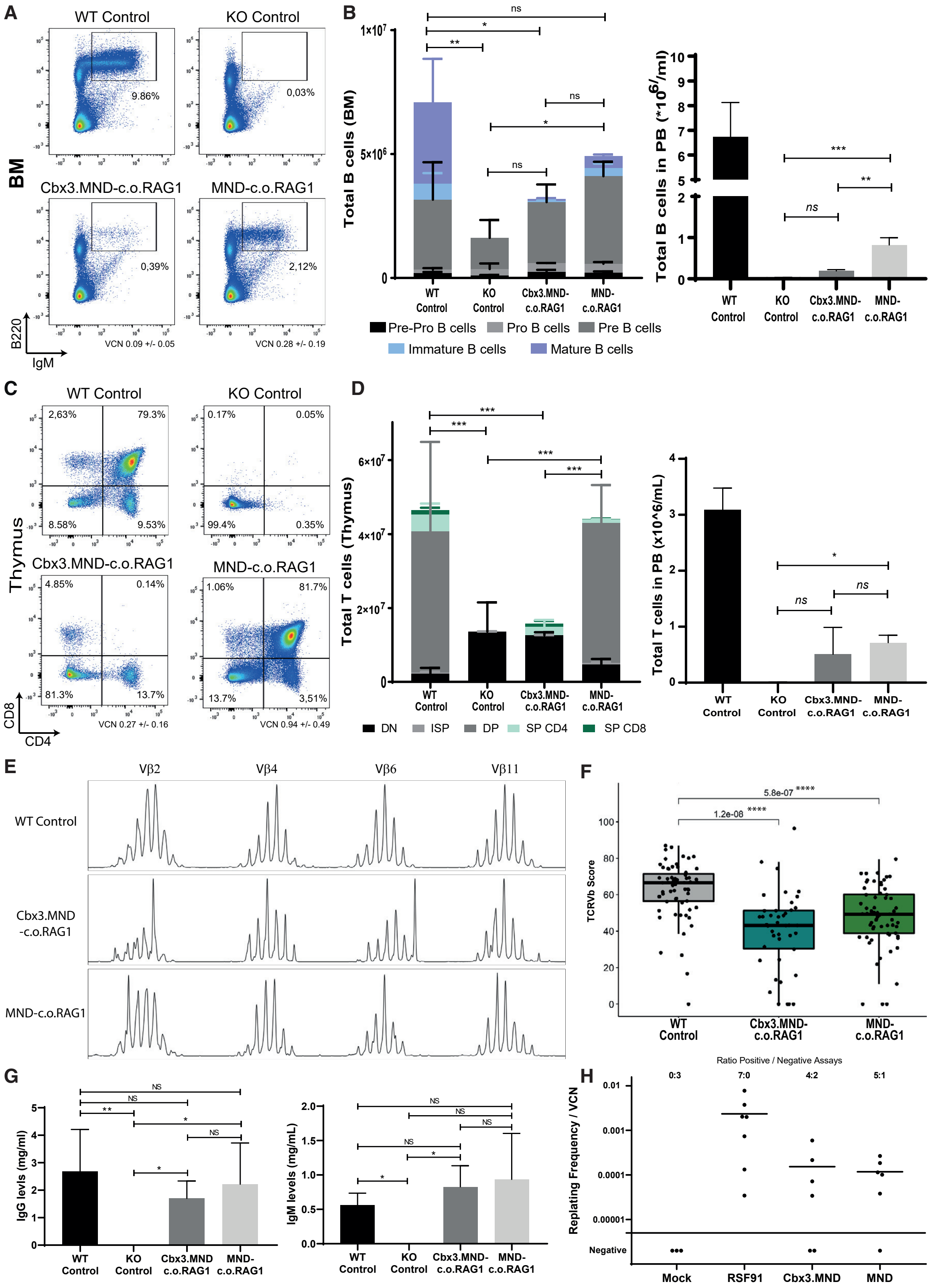

(legend on next page)

16 weeks after transplantation (Figures 2C and 2D). Similar to B cells, the total number of T cells in the periphery was lower than in mice treated with WT cells; nonetheless, mature T cells after gene therapy showed a diverse TCR repertoire. We used GeneScan analysis for 24 different Vβ genes and calculated the cumulative complexity score. As shown in the representative plots (Figure 2E) as well by the ImSpectR score (Figure 2F), the MND promoter performed closer to WT-treated mice, revealing an active V(D)J recombination machinery able to successfully rearrange *TCR* genes.

Besides efficacy, safety is an important aspect for clinical use of gene therapy vectors. As an additional selection criterion, our research grade lentivirus batches were tested in the *in vitro* immortalization (IVIM) assay, which is the currently accepted (US Food and Drug Administration [FDA] and European Medicines Agency [EMA] approved) standard assay for safety of viral vectors. Even though high VCNs per cells were achieved in this assay with the test vectors, both vectors were shown to have a frequency of insertional mutagenic events that were at least 50-fold lower than classical RSF91 γ-retroviral vectors with known mutagenic potential (Figure 2H). In three independent IVIM assays, we did not observe cytotoxicity of the vector supernatants on lineage-negative BM cells. This safety selection criterion, together with the successful *in vivo* immune reconstitution given by the MND-co.oRAG1-treated cells (Figure S1C), led us to conclude that the pCCL-MND-c.o.RAG1 LV vector is the best vector of choice, and we therefore proceeded to have the vector produced at good manufacturing practice (GMP) grade. All following experiments described were conducted with this clinical grade vector for further preclinical testing.

Extensive Preclinical Testing of the pCCL-MND-c.o.RAG1 LV Vector in *Rag1*$^{-/-}$ Mice

Initial analysis of eight *Rag1*$^{-/-}$ mice treated with the MND vector (starting VCN of 0.2), positive controls (WT stem cells; three mice), and negative controls (mock-transduced *Rag1*$^{-/-}$ stem cells; three mice) 24 weeks after transplantation confirmed good B cell reconstitution in the periphery (PB) and in BM (Figure 3A), although the numbers remained lower than mice treated with WT stem cells (Figure 3B; Figure S2A), which could be due to partially arrested development from pre-B to immature B cell stages (Figure S2B) originating from cells that were transduced with insufficient levels of *c.o.RAG1* to support full Ig rearrangements. Alternatively, residual progenitor (pro-) and pre-B cells could inhibit B cell development by occupying important developmental niches. However, gene therapy mice showed a similar proportion of immature and mature B cell subsets in the spleen (Figure 3C). Concerning T cell reconstitution, most gene therapy mice showed next to complete thymic T cell development with thymocyte numbers almost normal (Figure 3D; Figures S2A and S2B), although the T cell numbers in the periphery were restored to ~30% of normal levels (Figure 3E), with a somewhat lower proportion of naive CD4 and CD8 T cells and increased effector memory subsets (Figure 3F), most likely due to homeostatic proliferation from initial T cells that egressed from the thymus. Indeed, delayed T cell development can be observed in the gene therapy mice compared to WT controls (Figures S2A and S2B), and therefore the proportions of naive and memory T cells might still not be entirely balanced after gene therapy. Besides analyzing the primary and secondary immunological organs by flow cytometry, we also checked restoration of the immune system by histological analyses. Spleen, lymph nodes, and thymus showed remarkably normal architecture after gene therapy (Figure 3G), comparable to mice treated with WT stem cells, and quite different from the negative control mice treated with mock-transduced *Rag1*$^{-/-}$ cells. Importantly, restoration of FoxP3 expression, which directs T cells into the CD4$^+$ regulatory T cell (Treg) lineage, was also observed in mice treated with MND-c.o.RAG1 gene therapy (Figure 3G).

Functional Reconstitution of Immunity after RAG1 Gene Therapy

Next, we tested whether the T and B cells that developed had a diverse repertoire and were capable of mounting an immune response against a T cell-dependent neoantigen. GeneScan analysis (three WT control mice, one KO control mouse, and eight MND-c.o.RAG1 mice) showed a diverse TCR Vβ repertoire that was slightly less complex before immunization than in mice reconstituted with WT stem cells (Figure 4A), but after immunization there was no statistical difference in the immune repertoire. Total IgM, IgG, and IgE levels were also checked (Figure 4B; Figure S2D) and reached close to normal levels in gene therapy-treated mice. Therefore, although gene therapy mice were lagging behind with regard to B cell numbers, their functionality in the form of antibody production was restored to WT levels. We used 2,4,6-trinitrophenyl (TNP)-conjugated keyhole limpet hemocyanin (KLH) as T cell-specific antigen and measured

Figure 2. Selecting the Optimal SIN LV Plasmid to Drive an Immune Reconstitution of Rag1 Deficiency

Rag1-deficient mice (experiment with a total of 3 mice/group) were transplanted with 500,000 stem cells: WT cells, mock Rag1 KO cells, Cbx3.MND-c.o.RAG1-treated KO cells (VCN of 0.95), and MND-c.o.RAG1-treated KO cells (VCN of 1.1). (A) Representative FACS plots showing the restoration of B220^{high+} B cells in the BM. (B) Total number of the different B cell subsets in the BM (left panel) and total number of B cells (B220^{high+}) in the PB (right panel) 16 weeks after SC transplantation. Graphs represent the means and standard deviation of a pilot experiment with two to three mice per group (Mann-Whitney test, one-tailed; *p $\leq$ 0.05; NS, not significant). (C) Representative FACS plots of the thymus reconstitution (CD4 versus CD8) with the different constructs. (D) Total number of the different T cell subsets in the thymus (left panel) and total number of T cells (CD3$^+$TCRαβ$^+$) in PB (right panel) 16 weeks after transplantation. Graphs represent the means and standard deviation of a pilot experiment with two to three mice per group (Mann-Whitney test, one-tailed; *p $\leq$ 0.05; NS, not significant). DN, double negative; ISP, immature single positive; DP, double positive; SP, single positive. (E) Representative samples of GeneScan plots are shown for four different families (x axis indicates CDR3 length; y axis shows the fluorescence intensity of the runoff products). (F) TCR Vβ repertoire analysis by GeneScan. A total of 24 Vβ families were analyzed on spleen cells from three mice per group. Overall score of all of the families was calculated for the different constructs (Mann-Whitney test; p values are represented on the plot; ****p < 0.0001; NS, not significant). (G) Quantification of total IgG and IgM in mice serum by ELISA (one-way ANOVA test; *p < 0.05, **p < 0.01). (H) IVIM assay was performed on the two constructs to assess their safety (mock cells as negative control; RSF91 γ-retroviral vector as a positive control). Data show results from three complete IVIM assays.

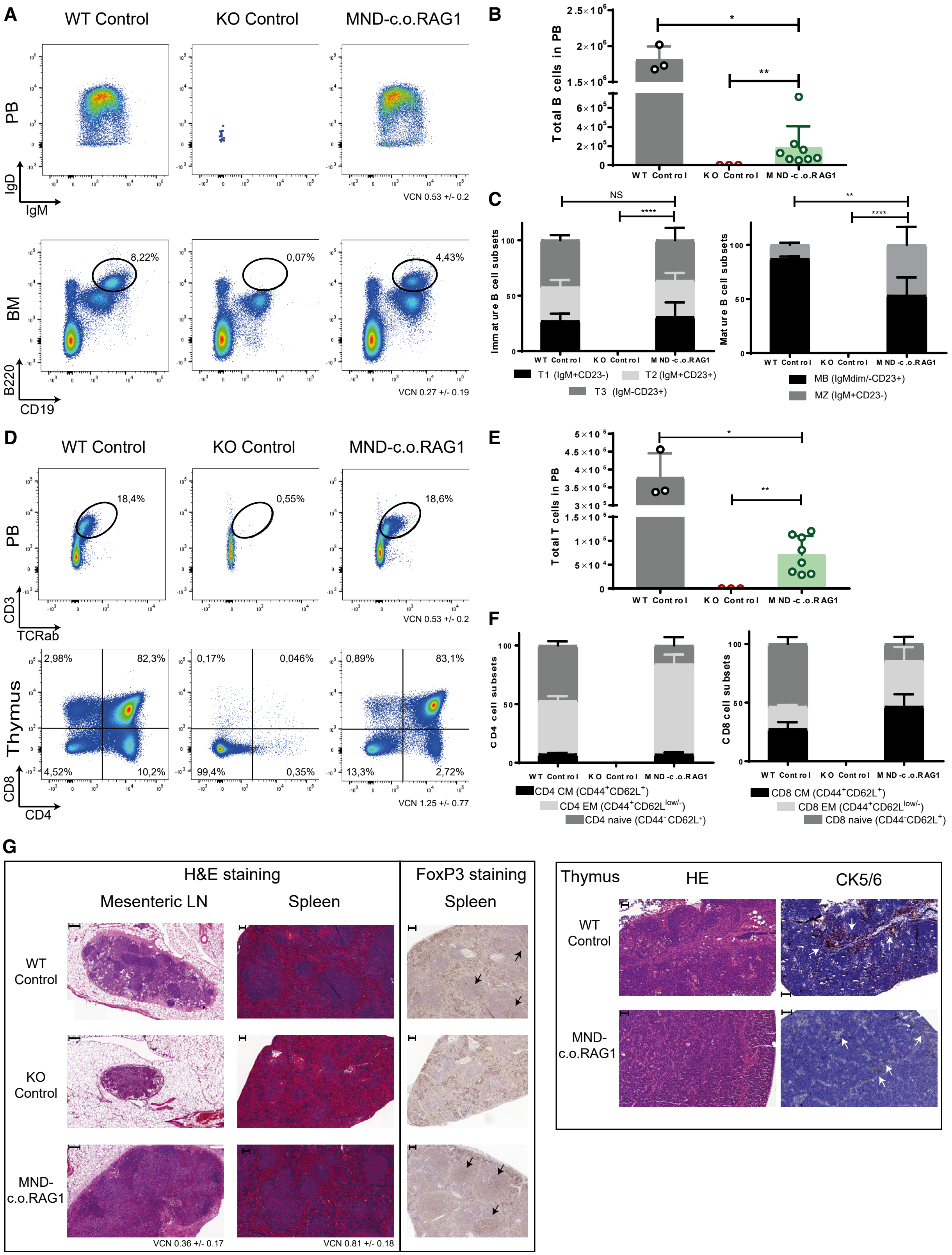

(legend on next page)

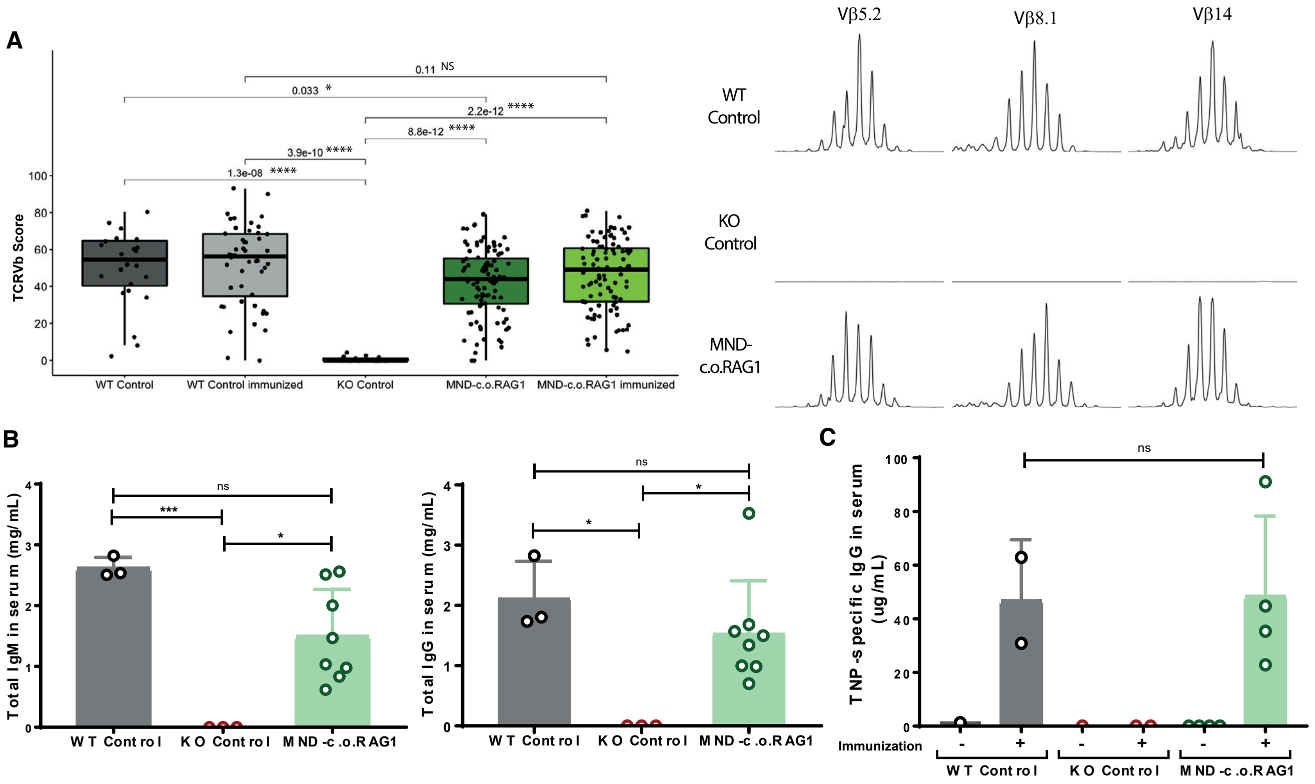

Figure 4. Functional Ig and TCR Rearrangements and Ig Class-Switch after RAG1 Gene Therapy

(A) TCR Vβ repertoire analysis by GeneScan from three WT control mice, one KO control mouse, and eight MND-c.o.RAG1 mice. A total of 24 Vβ families were analyzed on spleen cells from three WT control, one KO control, and eight MND-c.o.RAG1 mice (non-immunized and immunized). Overall score of all of the families was calculated by ImSpectR (Mann-Whitney test; p values are represented in the plot; $^{*}p < 0.05$, $^{****}p < 0.0001$; NS, not significant). Representative samples of GeneScan plots are shown for three different families (x axis indicates CDR3 length; y axis shows the fluorescence intensity of the runoff products). (B) Quantification of total IgG and IgM in serum by ELISA (three mice/control group, eight MND-c.o.RAG1 mice) (one-way ANOVA test; $^{*}p < 0.05$). (C) Quantification of TNP-specific IgG in serum of immunized mice. Each dot represents a value obtained in one mouse (three mice/control group, eight MND-c.o.RAG1 mice) (one-way ANOVA test; $^{*}p < 0.05$).

the production of TNP-specific IgG antibodies, thereby investigating whether the developed T and B cells could collaborate in an active immune response. The TNP-specific IgG levels in serum were similar between mice treated with WT stem cells and gene therapy-treated mice (Figure 4C), showing the potential of a robust immune response after gene therapy.

Preclinical Release Tests of the Vector

As required by regulatory authorities, the clinical grade vector was tested by external parties for the presence of replication-competent lentivirus (RCL). The vector tested negative in two independent tests (data not shown). Other release tests that are commonly required included biodistribution of the vector *in vivo*, checking of vector

Figure 3. Extensive Immune Reconstitution of Mice Receiving Gene Therapy of Stem Cells with a Clinical-Grade MND-c.o.RAG1 Vector

Rag1-deficient mice were transplanted with 250,000 stem cells: WT cells (three mice), mock KO cells (three mice), and MND-c.o.RAG1-treated cells (VCN of 0.2; eight mice). (A) Representative plots of B cell reconstitution in the blood ($B220^{+}$IgM/IgD cells; top panel) and B cell development in the BM ($B220^{+}CD19^{+}$ cells; bottom panel) 24 weeks after transplantation. (B) Total number of B cells ($B220^{+}CD11b/CD43^{-}$ cells) in the PB (Mann-Whitney test, one-tailed; $^{*}p < 0.05$, $^{**}p < 0.01$). (C) Immature ($B220^{+}CD93^{+}$ cells; left panel) and mature ($B220^{+}CD93^{-}$ cells; right panel) B cell subsets distribution in spleen. Two-way ANOVA test; $^{***}p < 0.001$; $^{****}p < 0.0001$. (D) Representative plots of T cell reconstitution in the blood ($CD3^{+}TCRab^{+}$ cells; top panel) and T cell development in the thymus (CD4 versus CD8 cells; bottom panel) 24 weeks after transplantation. (E) Total number of T cells ($CD3^{+}TCRab^{+}$ cells) in PB at the end of the experiment (24 weeks) (Mann-Whitney test, one-tailed; $^{*}p < 0.05$, $^{**}p < 0.01$). (F) Naive, effector memory (EM), and central memory (CM) subset distributions for CD4 ($CD3^{+}TCRab^{+}CD4^{+}$; left panel) and CD8 ($CD3^{+}TCRab^{+}CD8^{+}$; right panel). T cell subset distributions in spleen are shown: naive cells ($CD44^{-}CD62L^{+}$), EM cells ($CD44^{+}CD62L^{-}$) and CM cells ($CD44^{+}CD62L^{+}$) 24 weeks after transplantation. (G) Left panel: Hematoxylin and eosin staining of mesenteric lymph nodes (scale bars, 200 μm) and spleen (scale bars, 100 μm; purple indicates germinal centers, and red indicates red pulp). Representative FoxP3 staining in spleen tissue (scale bars, 100 μm) is shown. Arrows indicate positive FoxP3 in germinal centers. Representative images are from WT control, KO control, and MND-c.o.RAG1 gene therapy mice. Right panel: Histological analysis of thymus reconstitution by hematoxylin and eosin staining (scale bars, 50 μm) and cytokeratin 5/6 staining (scale bars, 100 μm) . Representative images from WT control and MND-c.o.RAG1 mice. KO thymus was completely used for phenotyping (FACS, DNA, RNA), but KO thymic histology was previously described by van Til et al.[27]

insertion sites, especially on possible clonal outgrowth, and tests for insertional mutagenesis such as IVIM.

We checked vector distribution on a large number of perfused organs (Table S1) in all gene therapy-treated mice (a total of eight mice; Figure 5A). Perfusion was used to remove most of the blood cells, in which the leukocytes should carry the vector. As expected, given the positive selection for c.o.RAG1-transduced cells, a high VCN was found in the thymus, followed by other immune organs, spleen, BM, lymph nodes, and PB. All other organs had very low signals, except some incidental positivity in stomach and lungs, possibly due to incomplete perfusion, or an ongoing infection in rare individual mice. Importantly, pathological examination of histology slides of 29 different organs per mouse (n = 14) did not show any abnormalities in mice treated with MND-c.o.RAG1 gene therapy (examples of four organs shown in Figure S2C). Indeed, no signs of Omenn syndrome such as skin rashes, high IgE levels, oligoclonal TCR Vβ repertoire, or T cell infiltrates in the skin were detected in the immune reconstituted mice.

Next, we checked viral insertion sites using non-restrictive linear amplification-mediated PCR (nrLAM-PCR) (Figure 5B), a sensitive technique that can detect clonal insertions as discrete bands, which can then be sequenced if needed.[34] We invariably found a smear of bands indicating polyclonal hematopoiesis with very little indication of oligoclonality, except for a few minor bands from which we could not get extra specific insertion site information by sequencing. We conclude that there was no evidence of vector-induced clonal selection. This is in line with findings by others on using SIN LV vectors in HSCs.

Safety of the clinical grade MND-c.o.RAG1 was also tested using the IVIM assay. The clinical vector showed no clonal outgrowth in different independent experiments, close to results from mock-transduced cells (Figure 5C). This is better than the research-grade vector presumably due to higher purity, resulting in a better functional titer and leading to fewer side effects after transduction.

Restored B and T Cell Development in RAG1-SCID Patient Cells

We have previously shown that transplantation of BM CD34$^+$ cells from SCID patients in NSG mice is informative for identifying where T cell development is arrested in human SCID.[8,35] This same model should also be suitable as a preclinical efficacy model with patient cells. Hence, we purified CD34$^+$ cells from cryopreserved BM cells from a RAG1-SCID patient. The patient was hypomorphic, with some residual B cells but no T cells. We transplanted busulfan-conditioned mice with either mock-transduced or MND-c.o.RAG1-transduced CD34$^+$ cells (one mouse per group; starting VCN of 0.2) and followed the development of T and B cells over time up to 24 weeks. Human cell engraftment was similar between mice transplanted with gene therapy-treated cells and mock-transduced cells, indicating that gene therapy did not affect the engraftment of human cells (Figure S3A). As expected from the patient phenotype, B cells were observed in the mock-transduced humanized mice, but much higher numbers of B cells were found in the spleen of the gene therapy-treated mice (Figure 6A; Figure S3B). The B cells that were present also showed polyclonal Ig rearrangement (Figure S3E) and produced Igs, as human IgM could be detected in the sera of the mice (Figure 6D), with a tendency toward a more polyclonal repertoire after gene therapy.

Importantly, while no T cells developed in the mouse transplanted with mock-transduced RAG1-SCID cells, the gene therapy mouse showed clearly detectable T cells in PB (Figure 6B; Figure S3C). After scarifying the mice, we also checked their thymi. As the patient was hypomorphic, we observed that some stages of T cell development were present, including all double-negative (DN; CD4$^-$CD8$^-$ cells), immature SP (ISP; CD4$^+$CD8$^-$CD3$^-$ cells), and the early CD3$^-$ DP stages (Figure 6C; Figure S3D). However, there were no cells that were CD3$^+$, and thus no late CD3$^+$ DP thymocytes or any SP thymocytes, suggesting that especially the rearrangement of TCRα was affected by this *RAG1* mutation. Although immune reconstitution was still not optimal, likely due to the low VCN achieved in that experiment, lentiviral RAG1 gene therapy of CD34$^+$ RAG1-SCID patient cells allows alleviation of the T cell developmental block and generation of an active thymus. Moreover, human cell engraftment and peripheral B and T cell levels after gene therapy were close to healthy BM CD34$^+$ cell transplantation described in previous work.[35] Finally, we checked *TCRB* and *TCRG* rearrangements by GeneScan analysis. Because of the very limited amount of DNA material, not all possible Vγ and Vβ genes could be analyzed, but the selected gene segments showed many more in-frame rearrangements in the gene therapy-treated group for *TCRG*, while for *TCRB* only in the gene therapy group, rearrangements could be detected (Figure 6E). nrLAM-PCR on BM cells revealed a polyclonal pattern with no signs of clonal dominance (Figure 6F).

DISCUSSION

Patients with RAG1-SCID are hampered in the genetic assembly of TCRs and BCRs. Affected children typically experience a wide range of serious, life-threatening infections. Replacing the affected BM with healthy, unmodified, allogeneic stem cells is currently the only therapy for RAG1-SCID. Although overall survival is satisfactory in matched-donor stem cell transplantation (SCT), the outcome in mismatched donor SCT, which represents most cases, is significantly worse. Moreover, approximately 25% of allogeneic SCT-treated patients develop graft-versus-host disease (GvHD), which significantly impairs outcome in terms of morbidity, immune reconstitution, and transplant-related mortality.[36] Additionally, transplant outcome in RAG-SCID (and other recombination-defective forms of T$^-$B$^-$ SCID) is significantly worse than for SCID with B cells (i.e., T$^-$B$^+$ SCID).[36,37]

Transplantation of genetically corrected autologous HSCs eliminates the risks associated with allogeneic SCT (GvHD and rejection) and would therefore provide a valuable alternative, particularly for patients lacking a matched donor. Gene therapy for X-linked SCID (X-SCID) with LV or retroviral SIN vectors has been shown to be

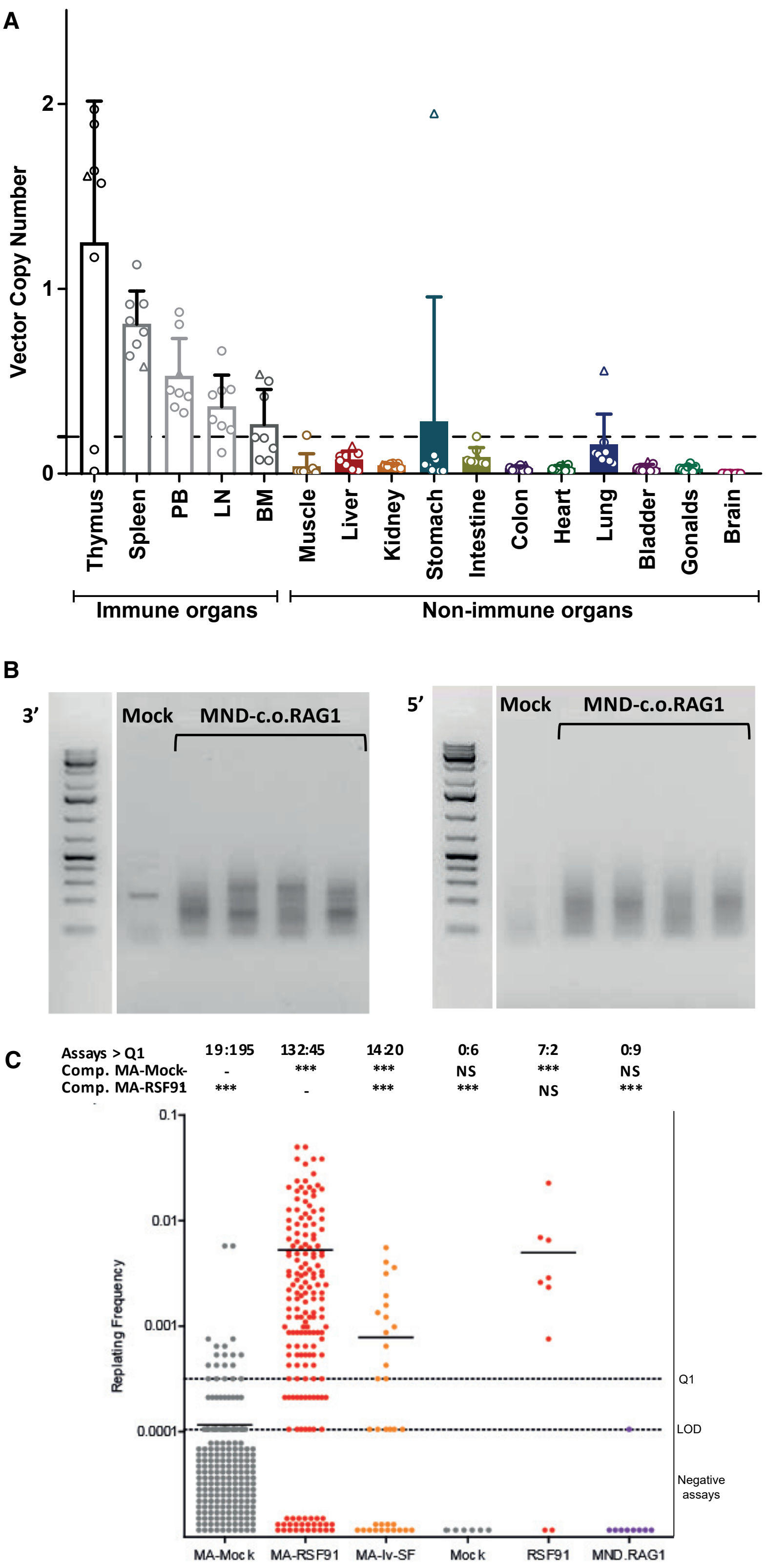

Figure 5. Preclinical Safety Testing of the Clinical-Grade MND-c.o.RAG1 Vector

(A) Vector biodistribution in immune and non-immune organs assessed by qPCR on DNA samples from 16 organs in total. Each dot represents a value from one mouse (three mice/control group, eight MND-c.o.RAG1 mice). The horizontal dashed line represents the threshold of the VCN of immune organs versus non-immune organs (starting VCN of transplanted cells of 0.2). (B) LV insertion site analysis by nrLAM-PCR of isolated DNA from BM obtained from *Rag1*$^{-/-}$ untransduced control mouse (mock) and four MND-c.o.RAG1 mice (male non-immunized/immunized, female non-immunized/immunized). Gels shows results of the linear amplification from the 3′ long terminal repeat (LTR) and 5′ LTR, respectively (L = 1 kb plus marker). (C) Replating frequencies (RFs) of the control samples mock or RSF91 and the test vector MND-c.o.RAG1, in comparison to data of a meta-analysis for control samples (Mock-MA, RSF91-MA, lv-SF-MA [a lentiviral vector with an SFFV promoter]). The data points below the limit of detection (LOD; plates with no wells above the MTT threshold) were manually inserted into the graph (due to the logarithmic scale of the y axis). Above the graph, the ratios of positive (left number) and negative plates (right number) according to the MTT assay are shown. Differences in the incidence of positive and negative assays relative to Mock-MA or RSF91-MA were analyzed by Fisher's exact test with a Benjamini-Hochberg correction (*$p < 0.05$, **$p < 0.01$, ***$p < 0.001$; NS, not significant). If above the LOD, bars indicate the mean RF.

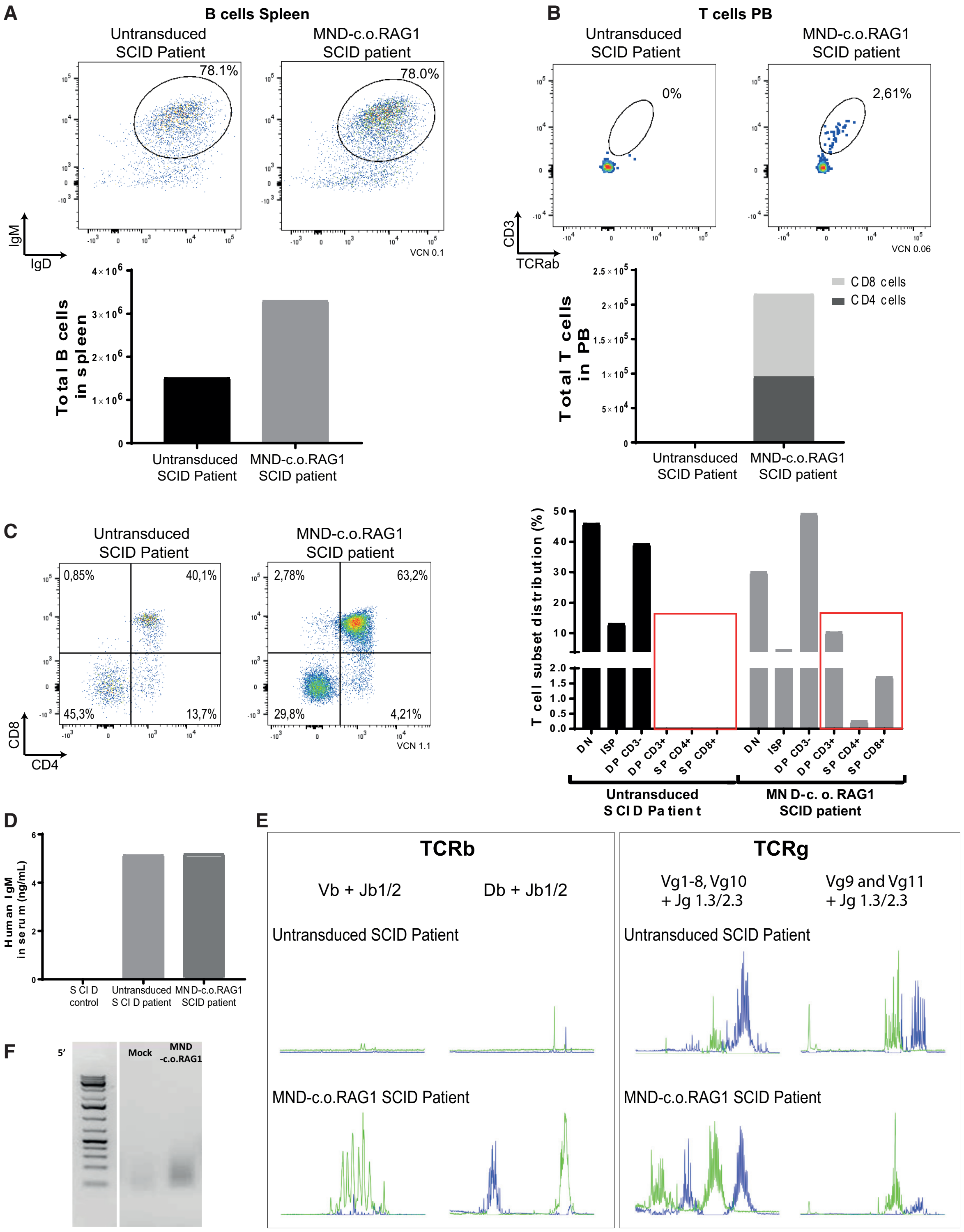

Figure 6. Restored T Cell Development in RAG1 SCID Patient Cells

65,000 human $CD34^+$ cells were transplanted intravenously into busulfan pre-conditioned NSG recipient mice (one NSG mouse with untreated cells and one NSG mouse with MND-c.o.RAG1 gene therapy cells with a VCN of 0.1). (A) FACS plots of human B cells ($CD13/33^-CD19^+CD20^+$ cells; top panel) and total number of B cells ($CD13/33^-CD19^+CD20^+$IgD/IgM cells; bottom panel) in the spleen at week 24 after transplantation. (B) FACS plots of human T cells ($CD3^+TCR\alpha\beta^+$; top panel) and total number of

(legend continued on next page)

successful and to lack the xenotoxicity problems previously observed when using γ-retroviral vectors.[38–40] For ADA-SCID, both retroviral vectors (currently marketed as approved therapy under the name Strimvelis) and LV vectors have shown excellent clinical results that are comparable to HSCT with matched donors.[10,41,42]

Unlike X-linked SCID and ADA-SCID, developing gene therapy for RAG-SCID has been notoriously difficult. Previous attempts[25] used γ-retroviral vectors in a preclinical $Rag1^{-/-}$ model, which carried a high risk of insertional mutagenesis. Although RAG1 γ-retroviral vectors were able to correct the deficiency more readily, SIN lentiviral vectors initially resulted in insufficient expression of the therapeutic *RAG1* gene, leading to "leaky" SCID or an Omenn-like phenotype. A breakthrough came with the introduction of codon optimization of the human *RAG1* gene.[26] This innovation yielded higher viral titers and much higher levels of *RAG1* expression without the need to introduce multiple copies per cell. In this study, we have used the same codon-optimized *RAG1* therapeutic gene, but in a different lentiviral backbone and under the control of a clinically approved promoter. The first challenge was to develop a vector with a strong promoter driving the high expression of *c.o.RAG1*, to similar levels as native expression. According to the ImmGen dataset and our previous data in human thymi,[33] native *Rag1* expression needed for B and T cell development in mouse is at least 10-fold and 13-fold that of the household gene expression (*Abl1*). In accordance, we show herein that durable, functional immune reconstitution can be obtained at low VCN (1 or lower) with our MND-c.o.RAG1 vector that is consistently driving sufficient *c.o.RAG1* expression above 10-fold that of the household gene. As proper *RAG1* expression was achieved, gene therapy-treated mice survived healthily, without showing representative features of leaky SCID in mice as discussed by Marrella et al.[43] (*Rag2* Omenn syndrome mouse model), Khiong et al.[44] (Rag1 Omenn syndrome), Giblin et al.[45] (atypical SCID phenotype), and Ott de Bruin et al.[46] (CID-G/AI [combined immunodeficiency with granuloma and/or autoimmunity] phenotype). Our data suggest that the approach using pCCL-MND-c.o.RAG1-transduced HSPCs should be able to overcome the broad range of clinical and immunologic phenotypes due to RAG1 deficiency, including hypomorphic RAG1 disease. Experimental proof for correction of hypomorphic RAG1 deficiencies requires extensive experimentation in appropriate mouse models, which are planned in the near future. Moreover, we show that the human RAG1 deficiency can be functionally restored in patient cells, providing important additional efficacy data required for successful clinical implementation.

In some mice, the reconstitution of T and B cell development with RAG1-transduced cells lagged behind compared to development observed in WT stem cells. This indicates that some additional improvements could be made, for example, by optimizing transduction efficiencies, which can be achieved by using a non-toxic transduction enhancer[47]; however, VCN numbers should not increase too much, as this may increase insertional mutagenic events. Another approach to improve at least the T cell development may be to co-transplant or to use $CD34^{+}CD7^{+}$ cells prior transplantation[48,49] from the same patient to support the thymic microenvironment in which the stem/progenitor cells that seed the thymus find their niches. This can be especially important to boost development in the DN compartment.

Insertional mutagenesis has been shown to occur in gene therapy trials using γ-retroviral vectors without SIN configuration. In our study a SIN LV vector using the MND promoter was chosen, because this fairly strong promoter is most efficacious in our preclinical models. The MND promoter has previously been used in gene therapy trials for ADA-SCID[50] and adrenoleukodystrophy (ALD), without any reports of insertional mutagenesis.[51,52] In the ALD trials there were some clones showing clonal dominance with over-representation of the insertion site near SMG6, CCND2, and HMGA2, but this has not led to development of leukemia and may be transient, as was reported for a SIN LV vector used for treating β-thalassemia.[53] In addition, our collective preclinical safety data indicate that the MND-c.o.RAG1 vector is relatively safe. Nevertheless, genotoxicity cannot be fully excluded and we therefore favor clinical implementation initially in patients in whom only histocompatibility leukocyte antigen (HLA)-incompatible donors are available. After clinical efficacy and safety has been demonstrated in this patient group, wider implementation could be considered, potentially not only for RAG1-SCID, but also for Omenn syndrome and other RAG1 deficiencies.

Clinical trials have shown that ADA-SCID and X-linked SCID gene therapies result in significant clinical benefit, as well as a significant reduction in healthcare-related costs (reviewed in Morgan et al.[50] and Staal et al.[54]). We expect similar benefits from our approach to treat patients with RAG1-SCID, as it will reduce the suboptimal outcomes in (mismatched) allogeneic transplants, which are often associated with the need to administer Igs, and treat infectious and GvHD-related complications. Based on the results reported hereinn, a phase I/II clinical trial is planned to open in 2020. We expect that this trial will provide an alternative curative treatment for patients with RAG1-SCID, for whom no matched stem cell donor is available.

T cells, CD4 cells, and CD8 T cells in the PB at week 24 after transplantation (bottom panel). (C) Human T cell development in the thymus: FACS plots (CD4 versus CD8) and distribution of the different T cells subsets in the thymus (24 weeks after transplantation) are shown. (D) Quantification of total human IgM by ELISA of serum from a control NSG mouse transplanted with RAG1-SCID control untreated $CD34^{+}$ cells (non-hypomorphic), our SCID patient $CD34^{+}$ cells, and our SCID MND-c.o.RAG1 $CD34^{+}$ cells. (E) Human TCR Vβ and Vγ repertoire analysis of isolated DNA from NSG thymus (SCID patient and SCID MND-c.o.RAG1) using a TCRB + TCRG T cell clonality assay (x axis indicates fragment sizes; y axis shows the fluorescence intensity of the runoff products). (F) LV insertion site analysis by nrLAM-PCR of isolated DNA from BM obtained from NSG SCID patient untransduced cells (mock) and NSG SCID MND-c.o.RAG1 mice. Gel shows results of the linear amplification from the 5′ LTR (L = 1 kb plus marker). Data are from an independent experiment with n = 1 per condition.

MATERIALS AND METHODS

Mice

C57BL/6 *Rag1*$^{-/-}$ mice were originally obtained from The Jackson Laboratory (USA). C57BL/6 WT mice and NSG (NOD.Cg-*Prkdc*^{scid} *Il2rg*^{tm1Wjl}/SzJ) mice were purchased from Charles River (France). Mice were bred and maintained in the animal facility of Leiden University Medical Center (LUMC). All animal experiments were approved by the Dutch Central Commission for Animal Experimentation (Centrale Commissie Dierproeven [CCD]).

Lentiviral Vectors and Vector Production

The *RAG1* gene sequence was optimized as described by Pike-Overzet et al.[26] Briefly, this resulted in 90% of the codons being adapted to the codon bias of *Homo sapiens* genes. Furthermore, the GC content was raised from 48% to 61% and the number of *cis*-acting motifs was reduced from 21 to 0. The optimized *RAG1* sequence was synthesized by GeneArt (Regensburg, Germany). c.o.RAG1 was cloned into self-inactivating lentiviral pCCL plasmid, resulting in pCCL-Cbx3. MND.coRAG1 (hereafter Cbx3.MND-c.o.RAG1), pCCL-MND-c.o.RAG1 (hereafter MND-c.o.RAG1), pCCL-PGK-c.o.RAG1 (hereafter PGK-c.o.RAG1), and pCCL-UCOE-c.o.RAG1 (hereafter UCOE-c.o.RAG1). DNA sequencing of the transgene was performed to validate the gene transfer constructs. Helper plasmids pMDLg/pRRE, pRSV-Rev, and pMD2.VSV-G for lentiviral production were kindly provided by L.Naldini (San Raffaele Telethon Institute for Gene Therapy, Milan, Italy).[30] Large-scale helper-plasmid preparations were obtained through PlasmidFactory (Bielefeld, Germany).

293T cells were transiently transfected with the transfer and helper plasmids using X-tremeGENE HP DNA transfection reagent (Sigma-Aldrich). Lentiviruses were harvested 24, 30, and 48 h after transfection, filtered through 0.22-μm pore filters (Whatman), and stored at −80°C. Pooled lentiviral supernatant was concentrated by ultracentrifugation (Beckman Optima LE-80K, rotor SW32Ti) for 16 h at 10,000 rpm and 4°C under vacuum conditions. Pellets were resuspended in StemSpan serum-free expansion medium (SFEM; STEMCELL Technologies) and aliquoted to avoid multiple freeze/thaw cycles. Since no suitable anti-RAG1 antibodies were available, we determined the viral titer using qPCR as described below. A clinical GMP-grade vector was generated by Batavia Biosciences (Leiden, the Netherlands), aliquoted in 200-μL vials, and stored at −80°C until use. The GMP-grade vector was tested and validated on murine Rag1-deficient BM cells, human CD34^{+} cells.

Transduction of Murine Lineage-Negative BM Cells and Human CD34^{+} Cells

Murine BM cells were obtained from femurs and tibias of C57BL/6 WT and C57BL/6 *Rag1*$^{-/-}$ mice. The obtained bones were flushed or crushed, and cells were passed through a 0.7-μm cell strainer (Falcon), washed, and viable frozen. After thawing, lineage-negative cells were isolated using a mouse lineage depletion kit and AutoMACS cell sorter (Miltenyi Biotec). Lineage-negative cells were stimulated overnight in StemSpan SFEM containing penicillin/streptomycin (5,000 U/5,000 μg/mL; Gibco) and supplemented with 50 ng/mL recombinant mouse FMS-related tyrosine kinase 3 ligand (rmFlt3L; R&D Systems), 100 ng/mL recombinant mouse stem-cell factor (rmSCF; R&D Systems), and 10 ng/mL recombinant mouse thrombopoietin (rmTPO; R&D systems). *Rag1*$^{-/-}$ cells were subsequently transduced with the different lentiviruses using 4 μg/mL protamine sulfate (Sigma-Aldrich) and by way of spin-occulation at 800 × *g* and 32°C for 1 h. Cells were cultured at 37°C, 5% CO_2 for 24 h in medium supplemented with cytokines.

Human BM from children diagnosed with SCID was obtained according to the Medical Ethical Committee and Institutional Review Board (IRB) guidelines at LUMC. The patient in this study was a compound heterozygote with the following confirmed mutations: RAG1 allele 1, C256–257 deletion A; allele 2, C1677G>T. Mononuclear cells were separated by Ficoll gradient centrifugation, frozen in fetal calf serum (Greiner Bio-One)/10% DMSO (Sigma-Aldrich), and stored in liquid nitrogen. After thawing, human CD34^{+} cells were isolated using CD34 a MicroBead Kit UltraPure (Miltenyi Biotec). Enriched CD34^{+} cells were stimulated overnight in X-VIVO15 without gentamycin and phenol red (Lonza)/1% human albumin (200 g/L; Sanquin)/penicillin/streptomycin medium supplemented with 300 ng/mL human stem cell factor (huSCF) (Miltenyi Biotec), 100 ng/mL human TPO (huTPO) (Miltenyi Biotec), 300 ng/mL human Flt3L (huFlt3L) (Miltenyi Biotec), and 10 ng/mL human interleukin 3 (huIL3) (Miltenyi Biotec). Cells were transduced in X-VIVO-15 complete medium with 4 μg/mL protamine sulfate as described previously and cultured for 24 h.

Transplantation of *Rag1*$^{-/-}$ and NSG Mice

Control mock-transduced cells (C57BL/6 WT cells, referred to as WT control, and *Rag1*$^{-/-}$ cells, referred to as KO control) and transduced *Rag1*$^{-/-}$ murine cells (equal amount of cells per group, up to 5×10^5 cells/mouse depending on the experiment) were mixed with supportive *Rag1*$^{-/-}$ spleen cells (3×10^6 cells/mouse) in Iscove's modified Dulbecco's medium (IMDM) without phenol red (Gibco) and transplanted by tail vein injection into preconditioned *Rag1*$^{-/-}$ recipient mice. Recipient mice (8–12 week old mice) were conditioned with total-body single-dose irradiation 24 h prior the transplantation using orthovoltage X-rays (8.08 Gy) or with two consecutive doses of 25 mg/kg busulfan (Sigma-Aldrich) (48 and 24 h prior to transplantation).

After overnight culture, 60,000–70,000 human CD34^{+} cells were resuspended in IMDM without phenol red (Gibco) and transplanted intravenously into busulfan pre-conditioned NSG recipient mice (5 weeks old mice, busulfan conditioning as described above).

Mice used for transplantation were kept in a specified pathogen-free section. The first 4 weeks after transplantation mice were fed with additional DietGel recovery food (ClearH_2O) and antibiotic water containing 0.07 mg/mL polymyxin B (Bupha Uitgeest), 0.0875 mg/mL ciprofloxacin (Bayer), and 0.1 mg/mL amphotericine B (Bristol-Myers Squibb) and their welfare was monitored daily. PB

from the mice was drawn by tail vein incision every 4 weeks until the end of the experiment. PB, thymus, spleen, and BM were obtained from CO_2 euthanized mice.

Immunization

Mice were immunized with synthetic TNP-KLH antigen 4 weeks before the end of the experiment. 100 μg of TNP-KLH (Biosearch Technologies) in 50% Imject alum (aluminum hydroxide) (Thermo Scientific) was injected intraperitoneally (i.p.). 3 weeks later, mice were boosted i.p. with 100 μg of TNP-KLH in PBS. Serum was collected before immunization and 1 week after the boost injection.

Flow Cytometry

Single-cell suspensions from thymus and spleen were prepared by squeezing the organs through a 70-μm cell strainer (BD Falcon), and a single-cell suspension from BM was made as described above. Erythrocytes from PB and spleen were lysed using NH_4Cl (8.4 g/L)/$KHCO_3$ (1 g/L) solution. Single-cell suspensions were counted and stained with the antibodies listed in Table S2. Briefly, cells were incubated for 30 min at 4°C in the dark with the antibody-mix solution including directly conjugated antibodies at the optimal working solution in fluorescence-activated cell sorting (FACS) buffer (PBS [pH 7.4], 0.1% sodium azide, 0.2% BSA). After washing with FACS buffer, a second 30-min incubation step at 4°C was performed with the streptavidin-conjugated antibody solution. When necessary, 7-aminoactinomycin D (7AAD) (BD Biosciences) was used as viability dye. Cells were measured on a FACSCanto II and LSRFortessa X-20 (BD Biosciences), and the data were analyzed using FlowJo software (Tree Star).

Determination of VCN and *c.o.RAG1* Expression by qPCR

Quantitative real-time PCR (qPCR) was used for the quantitative analysis of genomic lentiviral RNA, proviral DNA copies, and transgene mRNA expression using WPRE, c.o.RAG1, ABL, and PTBP2 as targets (Table S3). Total RNA from single-cell suspensions was purified using an RNeasy Mini kit (QIAGEN) and reverse transcribed into cDNA using a SuperScript III kit (Invitrogen). Genomic DNA was extracted from single-cell suspensions using a GeneElute mammalian genomic DNA kit (Sigma-Aldrich). A DNeasy blood and tissue kit (-QIAGEN) was used to isolate genomic DNA from murine organs and tissues. VCN was determined on DNA samples by the detection of WPRE and PTBP2. The levels of transgene expression were determined on cDNA samples by normalizing *c.o.RAG1* to the expression of the *ABL* gene. qPCR was performed using TaqMan universal master mix II (Thermo Fisher Scientific) in combination with specific probes for indicated genes from the Universal Probe Library (Roche). Primers and probes used are listed in Table S3. PCR reactions were performed on the StepOnePlus real-time PCR system (Thermo Fisher Scientific). All samples were run in triplicate.

Serum Ig Quantification

Murine IgG, IgM, IgE, TNP-specific IgG, and human IgM were determined by a sandwich enzyme-linked immunosorbent assay (ELISA). NUNC Maxisorp plates (Thermo Scientific) were coated with unlabeled anti-mouse IgG, IgM (11E10), IgE antibodies (SouthernBiotech), or unlabeled anti-human IgM antibody (Jackson ImmunoResearch Laboratories, kindly provided by Dr. Karahan, LUMC). For detection of TNP-specific IgG, plates were coated with synthetic TNP-KLH (Biosearch Technologies). Blocking was done with 1% BSA/PBS (mouse) or 2% BSA/0.025 Tween 20/PBS (human) for 1 h at room temperature (RT), and subsequently serial dilutions of the obtained sera were incubated for 3 h at RT. After washing, plates were incubated with biotin-conjugated anti-mouse IgG, IgM, IgE (SouthernBiotech), or anti-human IgM (Novex/Life Technologies, kindly provided by Dr. Karahan, LUMC) for 30 min at RT. For detection, plates were incubated for 30 min at RT with streptavidin horseradish peroxidase (Jackson ImmunoResearch Laboratories), and subsequently 2,2′-azino-bis(3-ethylbenzothiazoline-6-sulfonic acid) (ABTS) (Sigma-Aldrich) was used as a substrate. Data were acquired at a wavelength of 415 nm using a Bio-Rad iMark microplate reader and Microplate Manager Software 6 (MPM 6) (Bio-Rad). Antibody concentration was calculated by using serial dilutions of purified IgG, IgM, IgE proteins (SouthernBiotech), and human reference serum (Bethyl Laboratories, kindly provided by Dr. Karahan, LUMC) as standards.

Repertoire Analysis

Total RNA was purified from murine spleen cells and reverse transcribed into cDNA as described previously. The GeneScan analysis procedure of the murine T cell repertoire was adapted from Pannetier et al.[55] cDNA was amplified using a 6-carboxyfluorescein (FAM)-labeled C gene segment-specific primer along with 24 TCR Vβ-specific primers (see Table S3). GeneScan 500 ROX (Thermo Fisher Scientific) was used for internal size standard. Labeled PCR products were run on the ABI Prism genetic analyzer (Applied Biosystems) for fragment analysis. Raw spectratype data were analyzed, visualized, and scored by ImSpectR, a novel spectratype analysis algorithm for estimating immunodiversity.[56] ImSpectR identifies and scores individual spectratype peak patterns for overall (Gaussian) peak distribution, as well as the shape of individual peaks, while correcting for out-of-frame TCR transcripts. Scores range from 0, when no peaks are detected, to 100 for a diverse TCR repertoire.

The human Ig and TCR repertoire generated in NSG mice was analyzed on DNA samples from BM and thymus (DNA was extracted as described previously). Rearrangements were analyzed using the EuroClonality/BIOMED-2 multiplex PCR protocol.[57] Amplifications of IgH, IgK, TCRβ, and TCRγ rearrangements were performed following the *IGH* + *IGK* B cell clonality assay (Invivoscribe) and *TCRB* + *TCRG* T cell clonality assay (Invivoscribe) instructions, respectively. PCR products were analyzed by differential fluorescence detection using an ABI 3730 instrument (Applied Biosystems) for fragment analysis. The output files were visualized and analyzed using ImSpectR.

nrLAM-PCR

Lentiviral insertion site was analyzed by nrLAM-PCR on murine BM DNA samples as described by Schmidt and colleagues.[34]

IVIM Assay

The genotoxic potential of the viral vectors (Cbx3.MND-c.o.RAG1, MND-c.o.RAG1, PGK-c.o.RAG1, UCOE-c.o.RAG1) was quantified as previously described by Baum and colleagues.[58]

Gross Pathology and Histopathology

A full necropsy was performed, and organs were collected subjected to macroscopic and microscopic examination (see Table S1 for collected organs). The selection of organs to be examined for gross pathology and histopathology analyses followed the applicable European and international guidelines (EMEA 1995, World Health Organization [WHO] 2005).[59] For gross pathology, the external surface of the body, orifices, the thoracic abdominal and cavities were examined (analyzed organs are listed in Table S1).

For histopathological examination, organs were fixed in 4% neutral buffered formalin for 24 h and paraffin embedded; 5-μm sections were processed for hematoxylin and eosin (H&E) and immunohistochemistry staining according to standard procedures.[60] All slides were examined blindly by a European board certified pathologist (European College of Veterinary Pathologists [ECVP]).

Before staining, paraffin sections were deparaffinized. Antigen retrieval was performed for antibody against FOXP3 and cytokeratin 5/6 by heating during 12 min at 98°C in citric acid buffer (0.01 mol/L, pH 6.0). Inhibition of endogenous peroxidase was done in 0.3% H_2O_2 in PBS. After incubation overnight at RT with antibody against FOXP3 (1:70, 700914; Thermo Scientific, Waltham, MA, USA) and cytokeratin 5/6 (1:100, GA780; Dako, Glostrup, Denmark), the secondary antibody biotinylated goat anti-rabbit IgG (1:200, BA-1000; Vector Laboratories, Burlingame, CA, USA) and biotinylated horse anti-mouse (1:200, BA-2000; Vector Laboratories, Burlingame, CA, USA) was incubated for 90 min. Visualization was enforced with an ABC staining kit (Vectastain ABC kit, horseradish peroxidase [HRP], PK6100; Vector Laboratories, Burlingame, CA, USA) for 45 min. As substrate for HRP, 3,3′-diaminobenzidine tetrahydrochloride (DAB) (D5637; Sigma-Aldrich, St. Louis, MO, USA) was applied for 10 min. Mayer's hematoxylin was utilized for nuclear counterstaining.

Statistical Analysis

Statistics were calculated and graphs were generated using GraphPad Prism 6. Statistical significance was determined by a standard one- or two-tailed Mann-Whitney U test, an ANOVA test, or a two-tailed nonparametric Spearman correlation (*$p < 0.05$, **$p < 0.01$, ***$p < 0.001$, ****$p < 0.0001$).

SUPPLEMENTAL INFORMATION

Supplemental Information can be found online at https://doi.org/10.1016/j.omtm.2020.03.016.

AUTHOR CONTRIBUTIONS

L.G.-P., M.v.E., L.v.R., S.V., M.C., M.R., F.Z., D.S., and P.M. performed experiments. M.C., A.S., A.V., M.v.d.B., and J.J.M.V.D. helped in data analysis. D.B., A.S., M.C., A.J.T., C.L.P., J.J.M.V.D., J.-J.Z., H.B.G., and A.L. provided discussion on experiments aimed at clinical implementation and overall study design. F.J.T.S. and K.P.-O. designed experiments and the overall study concept. L.G.-P., A.L., F.J.T.S., and K.P.-O. wrote the manuscript.

CONFLICTS OF INTEREST

The authors declare no competing interests.

ACKNOWLEDGMENTS

This work was supported in part by ZonMW E-RARE grant 40-419000-98-020, EU H2020 grants SCIDNET (SEP-210221188) and RECOMB (755170-2), and has received funding from the European Union Horizon 2020 research and innovation program. A.J.T. and F.Z. were supported by the Wellcome Trust (104807/Z/14/Z). A.J.T., H.B.G., and F.Z. were supported by National Institute for Health Research Biomedical Research Centre at Great Ormond Street Hospital for Children NHS Foundation Trust/University College London Hospitals NHS Foundation Trust and University College London. We thank Drs. Willem Fibbe and Henk-Jan Guchelaar for continuous support. Dr. Isabelle Andre-Schmutz (Paris) is acknowledged for critically reading the manuscript and providing practical advice.

REFERENCES

1. Fischer, A., Hacein-Bey-Abina, S., and Cavazzana-Calvo, M. (2010). 20 years of gene therapy for SCID. Nat. Immunol. *11*, 457–460.

2. Griffith, L.M., Cowan, M.J., Notarangelo, L.D., Puck, J.M., Buckley, R.H., Candotti, F., Conley, M.E., Fleisher, T.A., Gaspar, H.B., Kohn, D.B., et al. (2009). Improving cellular therapy for primary immune deficiency diseases: recognition, diagnosis, and management. J. Allergy Clin. Immunol. *124*, 1152–1160.e12.

3. Grunebaum, E., Mazzolari, E., Porta, F., Dallera, D., Atkinson, A., Reid, B., Notarangelo, L.D., and Roifman, C.M. (2006). Bone marrow transplantation for severe combined immune deficiency. JAMA *295*, 508–518.

4. Cavazzana-Calvo, M., and Fischer, A. (2007). Gene therapy for severe combined immunodeficiency: are we there yet? J. Clin. Invest. *117*, 1456–1465.

5. Gaspar, H.B., and Thrasher, A.J. (2005). Gene therapy for severe combined immunodeficiencies. Expert Opin. Biol. Ther. *5*, 1175–1182.

6. Noordzij, J.G., de Bruin-Versteeg, S., Verkaik, N.S., Vossen, J.M., de Groot, R., Bernatowska, E., Langerak, A.W., van Gent, D.C., and van Dongen, J.J. (2002). The immunophenotypic and immunogenotypic B-cell differentiation arrest in bone marrow of RAG-deficient SCID patients corresponds to residual recombination activities of mutated RAG proteins. Blood *100*, 2145–2152.

7. Romano, R., Palamaro, L., Fusco, A., Iannace, L., Maio, S., Vigliano, I., Giardino, G., and Pignata, C. (2012). From murine to human nude/SCID: the thymus, T-cell development and the missing link. Clin. Dev. Immunol. *2012*, 467101.

8. Wiekmeijer, A.S., Pike-Overzet, K., IJspeert, H., Brugman, M.H., Wolvers-Tettero, I.L., Lankester, A.C., Bredius, R.G., van Dongen, J.J., Fibbe, W.E., Langerak, A.W., et al. (2016). Identification of checkpoints in human T-cell development using severe combined immunodeficiency stem cells. J. Allergy Clin. Immunol. *137*, 517–526.

9. Aiuti, A. (2002). Advances in gene therapy for ADA-deficient SCID. Curr. Opin. Mol. Ther. *4*, 515–522.

10. Aiuti, A., Cassani, B., Andolfi, G., Mirolo, M., Biasco, L., Recchia, A., Urbinati, F., Valacca, C., Scaramuzza, S., Aker, M., et al. (2007). Multilineage hematopoietic reconstitution without clonal selection in ADA-SCID patients treated with stem cell gene therapy. J. Clin. Invest. *117*, 2233–2240.

11. Aiuti, A., Cattaneo, F., Galimberti, S., Benninghoff, U., Cassani, B., Callegaro, L., Scaramuzza, S., Andolfi, G., Mirolo, M., Brigida, I., et al. (2009). Gene therapy for

immunodeficiency due to adenosine deaminase deficiency. N. Engl. J. Med. *360*, 447–458.

12. Carlucci, F., Tabucchi, A., Aiuti, A., Rosi, F., Floccari, F., Pagani, R., and Marinello, E. (2004). Evaluation of ADA gene expression and transduction efficiency in ADA/SCID patients undergoing gene therapy. Nucleosides Nucleotides Nucleic Acids *23*, 1245–1248.

13. Cavazzana-Calvo, M., Hacein-Bey, S., de Saint Basile, G., Gross, F., Yvon, E., Nusbaum, P., Selz, F., Hue, C., Certain, S., Casanova, J.L., et al. (2000). Gene therapy of human severe combined immunodeficiency (SCID)-X1 disease. Science *288*, 669–672.

14. Gaspar, H.B., Parsley, K.L., Howe, S., King, D., Gilmour, K.C., Sinclair, J., Brouns, G., Schmidt, M., Von Kalle, C., Barington, T., et al. (2004). Gene therapy of X-linked severe combined immunodeficiency by use of a pseudotyped gammaretroviral vector. Lancet *364*, 2181–2187.

15. Santilli, G., Thornhill, S.I., Kinnon, C., and Thrasher, A.J. (2008). Gene therapy of inherited immunodeficiencies. Expert Opin. Biol. Ther. *8*, 397–407.

16. Hacein-Bey-Abina, S., Von Kalle, C., Schmidt, M., McCormack, M.P., Wulffraat, N., Leboulch, P., Lim, A., Osborne, C.S., Pawliuk, R., Morillon, E., et al. (2003). *LMO2*-associated clonal T cell proliferation in two patients after gene therapy for SCID-X1. Science *302*, 415–419.

17. Kohn, D.B., Sadelain, M., and Glorioso, J.C. (2003). Occurrence of leukaemia following gene therapy of X-linked SCID. Nat. Rev. Cancer *3*, 477–488.

18. Pike-Overzet, K., de Ridder, D., Weerkamp, F., Baert, M.R., Verstegen, M.M., Brugman, M.H., Howe, S.J., Reinders, M.J., Thrasher, A.J., Wagemaker, G., et al. (2006). Gene therapy: is *IL2RG* oncogenic in T-cell development? Nature *443*, E5.

19. Pike-Overzet, K., van der Burg, M., Wagemaker, G., van Dongen, J.J., and Staal, F.J. (2007). New insights and unresolved issues regarding insertional mutagenesis in X-linked SCID gene therapy. Mol. Ther. *15*, 1910–1916.

20. Howe, S.J., Mansour, M.R., Schwarzwaelder, K., Bartholomae, C., Hubank, M., Kempski, H., Brugman, M.H., Pike-Overzet, K., Chatters, S.J., de Ridder, D., et al. (2008). Insertional mutagenesis combined with acquired somatic mutations causes leukemogenesis following gene therapy of SCID-X1 patients. J. Clin. Invest. *118*, 3143–3150.

21. Montini, E., Cesana, D., Schmidt, M., Sanvito, F., Bartholomae, C.C., Ranzani, M., Benedicenti, F., Sergi, L.S., Ambrosi, A., Ponzoni, M., et al. (2009). The genotoxic potential of retroviral vectors is strongly modulated by vector design and integration site selection in a mouse model of HSC gene therapy. J. Clin. Invest. *119*, 964–975.

22. Thornhill, S.I., Schambach, A., Howe, S.J., Ulaganathan, M., Grassman, E., Williams, D., Schiedlmeier, B., Sebire, N.J., Gaspar, H.B., Kinnon, C., et al. (2008). Self-inactivating gammaretroviral vectors for gene therapy of X-linked severe combined immunodeficiency. Mol. Ther. *16*, 590–598.

23. Benjelloun, F., Garrigue, A., Demerens-de Chappedelaine, C., Soulas-Sprauel, P., Malassis-Séris, M., Stockholm, D., Hauer, J., Blondeau, J., Rivière, J., Lim, A., et al. (2008). Stable and functional lymphoid reconstitution in Artemis-deficient mice following lentiviral Artemis gene transfer into hematopoietic stem cells. Mol. Ther. *16*, 1490–1499.

24. Lagresle-Peyrou, C., Yates, F., Malassis-Séris, M., Hue, C., Morillon, E., Garrigue, A., Liu, A., Hajdari, P., Stockholm, D., Danos, O., et al. (2006). Long-term immune reconstitution in RAG-1-deficient mice treated by retroviral gene therapy: a balance between efficiency and toxicity. Blood *107*, 63–72.

25. Lagresle-Peyrou, C., Benjelloun, F., Hue, C., Andre-Schmutz, I., Bonhomme, D., Forveille, M., Beldjord, K., Hacein-Bey-Abina, S., De Villartay, J.P., Charneau, P., et al. (2008). Restoration of human B-cell differentiation into NOD-SCID mice engrafted with gene-corrected $CD34^+$ cells isolated from Artemis or RAG1-deficient patients. Mol. Ther. *16*, 396–403.

26. Pike-Overzet, K., Rodijk, M., Ng, Y.Y., Baert, M.R., Lagresle-Peyrou, C., Schambach, A., Zhang, F., Hoeben, R.C., Hacein-Bey-Abina, S., Lankester, A.C., et al. (2011). Correction of murine Rag1 deficiency by self-inactivating lentiviral vector-mediated gene transfer. Leukemia *25*, 1471–1483.

27. van Til, N.P., Sarwari, R., Visser, T.P., Hauer, J., Lagresle-Peyrou, C., van der Velden, G., Malshetty, V., Cortes, P., Jollet, A., Danos, O., et al. (2014). Recombination-activating gene 1 (*Rag1*)-deficient mice with severe combined immunodeficiency treated with lentiviral gene therapy demonstrate autoimmune Omenn-like syndrome. J. Allergy Clin. Immunol. *133*, 1116–1123.

28. Pike-Overzet, K., Baum, C., Bredius, R.G., Cavazzana, M., Driessen, G.J., Fibbe, W.E., Gaspar, H.B., Hoeben, R.C., Lagresle-Peyrou, C., Lankester, A., et al. (2014). Successful RAG1-SCID gene therapy depends on the level of RAG1 expression. J. Allergy Clin. Immunol. *134*, 242–243.

29. Baum, C., Kustikova, O., Modlich, U., Li, Z., and Fehse, B. (2006). Mutagenesis and oncogenesis by chromosomal insertion of gene transfer vectors. Hum. Gene Ther. *17*, 253–263.

30. Dull, T., Zufferey, R., Kelly, M., Mandel, R.J., Nguyen, M., Trono, D., and Naldini, L. (1998). A third-generation lentivirus vector with a conditional packaging system. J. Virol. *72*, 8463–8471.

31. Halene, S., Wang, L., Cooper, R.M., Bockstoce, D.C., Robbins, P.B., and Kohn, D.B. (1999). Improved expression in hematopoietic and lymphoid cells in mice after transplantation of bone marrow transduced with a modified retroviral vector. Blood *94*, 3349–3357.

32. Knight, S., Zhang, F., Mueller-Kuller, U., Bokhoven, M., Gupta, A., Broughton, T., Sha, S., Antoniou, M.N., Brendel, C., Grez, M., et al. (2012). Safer, silencing-resistant lentiviral vectors: optimization of the ubiquitous chromatin-opening element through elimination of aberrant splicing. J. Virol. *86*, 9088–9095.

33. Dik, W.A., Pike-Overzet, K., Weerkamp, F., de Ridder, D., de Haas, E.F., Baert, M.R., van der Spek, P., Koster, E.E., Reinders, M.J., van Dongen, J.J., et al. (2005). New insights on human T cell development by quantitative T cell receptor gene rearrangement studies and gene expression profiling. J. Exp. Med. *201*, 1715–1723.

34. Gabriel, R., Kutschera, I., Bartholomae, C.C., von Kalle, C., and Schmidt, M. (2014). Linear amplification mediated PCR—localization of genetic elements and characterization of unknown flanking DNA. J. Vis. Exp. (88), e51543.

35. Wiekmeijer, A.S., Pike-Overzet, K., Brugman, M.H., Salvatori, D.C., Egeler, R.M., Bredius, R.G., Fibbe, W.E., and Staal, F.J. (2014). Sustained engraftment of cryopreserved human bone marrow $CD34^+$ cells in young adult NSG mice. Biores. Open Access *3*, 110–116.

36. Gennery, A.R., Slatter, M.A., Grandin, L., Taupin, P., Cant, A.J., Veys, P., Amrolia, P.J., Gaspar, H.B., Davies, E.G., Friedrich, W., et al. (2010). Transplantation of hematopoietic stem cells and long-term survival for primary immunodeficiencies in Europe: entering a new century, do we do better? J. Allergy Clin. Immunol. *126*, 602–610.e1–11.

37. Haddad, E., Logan, B.R., Griffith, L.M., Buckley, R.H., Parrott, R.E., Prockop, S.E., Small, T.N., Chaisson, J., Dvorak, C.C., Murnane, M., et al. (2018). SCID genotype and 6-month posttransplant CD4 count predict survival and immune recovery. Blood *132*, 1737–1749.

38. De Ravin, S.S., Wu, X., Moir, S., Anaya-O'Brien, S., Kwatemaa, N., Littel, P., Theobald, N., Choi, U., Su, L., Marquesen, M., et al. (2016). Lentiviral hematopoietic stem cell gene therapy for X-linked severe combined immunodeficiency. Sci. Transl. Med. *8*, 335ra57.

39. Touzot, F., Moshous, D., Creidy, R., Neven, B., Frange, P., Cros, G., Caccavelli, L., Blondeau, J., Magnani, A., Luby, J.M., et al. (2015). Faster T-cell development following gene therapy compared with haploidentical HSCT in the treatment of SCID-X1. Blood *125*, 3563–3569.

40. Cavazzana, M., Six, E., Lagresle-Peyrou, C., André-Schmutz, I., and Hacein-Bey-Abina, S. (2016). Gene therapy for X-linked severe combined immunodeficiency: where do we stand? Hum. Gene Ther. *27*, 108–116.

41. Mortellaro, A., Hernandez, R.J., Guerrini, M.M., Carlucci, F., Tabucchi, A., Ponzoni, M., Sanvito, F., Doglioni, C., Di Serio, C., Biasco, L., et al. (2006). Ex vivo gene therapy with lentiviral vectors rescues adenosine deaminase (ADA)-deficient mice and corrects their immune and metabolic defects. Blood *108*, 2979–2988.

42. Kohn, D.B., and Kuo, C.Y. (2017). New frontiers in the therapy of primary immunodeficiency: From gene addition to gene editing. J. Allergy Clin. Immunol. *139*, 726–732.

43. Marrella, V., Poliani, P.L., Casati, A., Rucci, F., Frascoli, L., Gougeon, M.L., Lemercier, B., Bosticardo, M., Ravanini, M., Battaglia, M., et al. (2007). A hypomorphic R229Q *Rag2* mouse mutant recapitulates human Omenn syndrome. J. Clin. Invest. *117*, 1260–1269.

44. Khiong, K., Murakami, M., Kitabayashi, C., Ueda, N., Sawa, S., Sakamoto, A., Kotzin, B.L., Rozzo, S.J., Ishihara, K., Verella-Garcia, M., et al. (2007). Homeostatically proliferating CD4 T cells are involved in the pathogenesis of an Omenn syndrome murine model. J. Clin. Invest. *117*, 1270–1281.

45. Giblin, W., Chatterji, M., Westfield, G., Masud, T., Theisen, B., Cheng, H.L., DeVido, J., Alt, F.W., Ferguson, D.O., Schatz, D.G., and Sekiguchi, J. (2009). Leaky severe combined immunodeficiency and aberrant DNA rearrangements due to a hypomorphic RAG1 mutation. Blood *113*, 2965–2975.

46. Ott de Bruin, L.M., Bosticardo, M., Barbieri, A., Lin, S.G., Rowe, J.H., Poliani, P.L., Ching, K., Eriksson, D., Landegren, N., Kämpe, O., et al. (2018). Hypomorphic *Rag1* mutations alter the preimmune repertoire at early stages of lymphoid development. Blood *132*, 281–292.

47. Delville, M., Soheili, T., Bellier, F., Durand, A., Denis, A., Lagresle-Peyrou, C., Cavazzana, M., Andre-Schmutz, I., and Six, E. (2018). A nontoxic transduction enhancer enables highly efficient lentiviral transduction of primary murine t cells and hematopoietic stem cells. Mol. Ther. Methods Clin. Dev. *10*, 341–347.

48. Six, E.M., Benjelloun, F., Garrigue, A., Bonhomme, D., Morillon, E., Rouiller, J., Cacavelli, L., Blondeau, J., Beldjord, K., Hacein-Bey-Abina, S., et al. (2011). Cytokines and culture medium have a major impact on human in vitro T-cell differentiation. Blood Cells Mol. Dis. *47*, 72–78.

49. Reimann, C., Six, E., Dal-Cortivo, L., Schiavo, A., Appourchaux, K., Lagresle-Peyrou, C., de Chappedelaine, C., Ternaux, B., Coulombel, L., Beldjord, K., et al. (2012). Human T-lymphoid progenitors generated in a feeder-cell-free Delta-like-4 culture system promote T-cell reconstitution in NOD/SCID/$\gamma c^{-/-}$ mice. Stem Cells *30*, 1771–1780.

50. Morgan, R.A., Gray, D., Lomova, A., and Kohn, D.B. (2017). Hematopoietic stem cell gene therapy: progress and lessons learned. Cell Stem Cell *21*, 574–590.

51. Eichler, F., Duncan, C., Musolino, P.L., Orchard, P.J., De Oliveira, S., Thrasher, A.J., Armant, M., Dansereau, C., Lund, T.C., Miller, W.P., et al. (2017). Hematopoietic stem-cell gene therapy for cerebral adrenoleukodystrophy. N. Engl. J. Med. *377*, 1630–1638.

52. Cartier, N., Hacein-Bey-Abina, S., Bartholomae, C.C., Veres, G., Schmidt, M., Kutschera, I., Vidaud, M., Abel, U., Dal-Cortivo, L., Caccavelli, L., et al. (2009). Hematopoietic stem cell gene therapy with a lentiviral vector in X-linked adrenoleukodystrophy. Science *326*, 818–823.

53. Cavazzana-Calvo, M., Payen, E., Negre, O., Wang, G., Hehir, K., Fusil, F., Down, J., Denaro, M., Brady, T., Westerman, K., et al. (2010). Transfusion independence and *HMGA2* activation after gene therapy of human β-thalassaemia. Nature *467*, 318–322.

54. Staal, F.J.T., Aiuti, A., and Cavazzana, M. (2019). Autologous stem-cell-based gene therapy for inherited disorders: state of the art and perspectives. Front Pediatr. *7*, 443.

55. Pannetier, C., Cochet, M., Darche, S., Casrouge, A., Zöller, M., and Kourilsky, P. (1993). The sizes of the CDR3 hypervariable regions of the murine T-cell receptor beta chains vary as a function of the recombined germ-line segments. Proc. Natl. Acad. Sci. USA *90*, 4319–4323.

56. Cordes, M., Pike-Overzet, K., van Eggermond, M., Vloemans, S., Baert, M.R., Garcia-Perez, L., Staal, F.J.T., Reinders, M.J.T., and van den Akker, E. (2020). ImSpectR: R package to quantify immune repertoire diversity in spectratype and repertoire sequencing data. Bioinformatics *36*, 1930–1932.

57. van Dongen, J.J.M., Langerak, A.W., Brüggemann, M., Evans, P.A., Hummel, M., Lavender, F.L., Delabesse, E., Davi, F., Schuuring, E., García-Sanz, R., et al. (2003). Design and standardization of PCR primers and protocols for detection of clonal immunoglobulin and T-cell receptor gene recombinations in suspect lymphoproliferations: report of the BIOMED-2 Concerted Action BMH4-CT98-3936. Leukemia *17*, 2257–2317.

58. Modlich, U., Bohne, J., Schmidt, M., von Kalle, C., Knöss, S., Schambach, A., and Baum, C. (2006). Cell-culture assays reveal the importance of retroviral vector design for insertional genotoxicity. Blood *108*, 2545–2553.

59. World Health Organization (2005). WHO Guidelines on Nonclinical Evaluation of Vaccines. WHO Technical Report Series No. 927 (World Health Organization), pp. 31–63.

60. Bancroft, J.D., and Gamble, M. (2008). Theory and Practice of Histological Techniques (Churchill Livingstone).

OMTM, Volume *17*

Supplemental Information

Successful Preclinical Development of Gene Therapy for Recombinase-Activating Gene-1-Deficient SCID

Laura Garcia-Perez, Marja van Eggermond, Lieke van Roon, Sandra A. Vloemans, Martijn Cordes, Axel Schambach, Michael Rothe, Dagmar Berghuis, Chantal Lagresle-Peyrou, Marina Cavazzana, Fang Zhang, Adrian J. Thrasher, Daniela Salvatori, Pauline Meij, Anna Villa, Jacques J.M. Van Dongen, Jaap-Jan Zwaginga, Mirjam van der Burg, H. Bobby Gaspar, Arjan Lankester, Frank J.T. Staal, and Karin Pike-Overzet

Suppl. Figure 1: Optimal SIN LV choice

A)

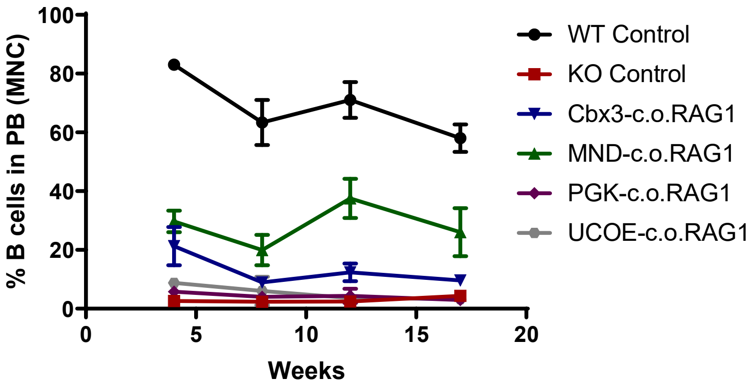

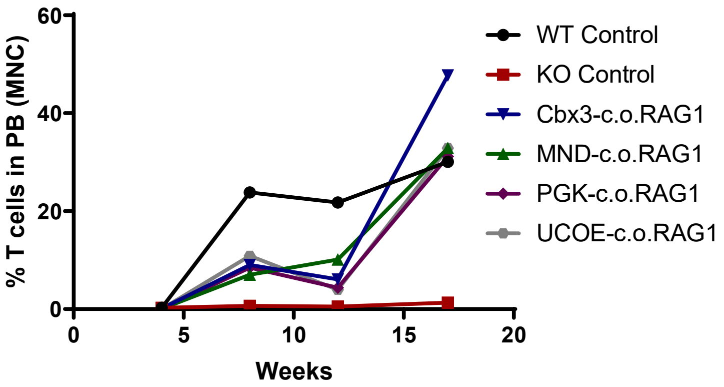

B)

In vitro	Cbx3-MND	MND	PGK	UCOE
Physical titer	++	++	+	+/-
Functional titer	+	++	+	+/-
Transgene expression	+	+	-	-
Promoter strength	++	+	-	+

C)

In vivo	Cbx3-MND	MND
B cell development BM	-	+
Thymic reconstitution	-	+
Mature immune cells	-	+
Immunoglobulins	-	+
T cell repertoire	+/-	+/-
Safety (IVIM)	+/-	+/-
Omenn-like syndrome in mice (due to low c.o.RAG1 expression)	Observed	Not observed

Suppl. Figure 2: Immune development after gene therapy in Rag1-/- mouse model

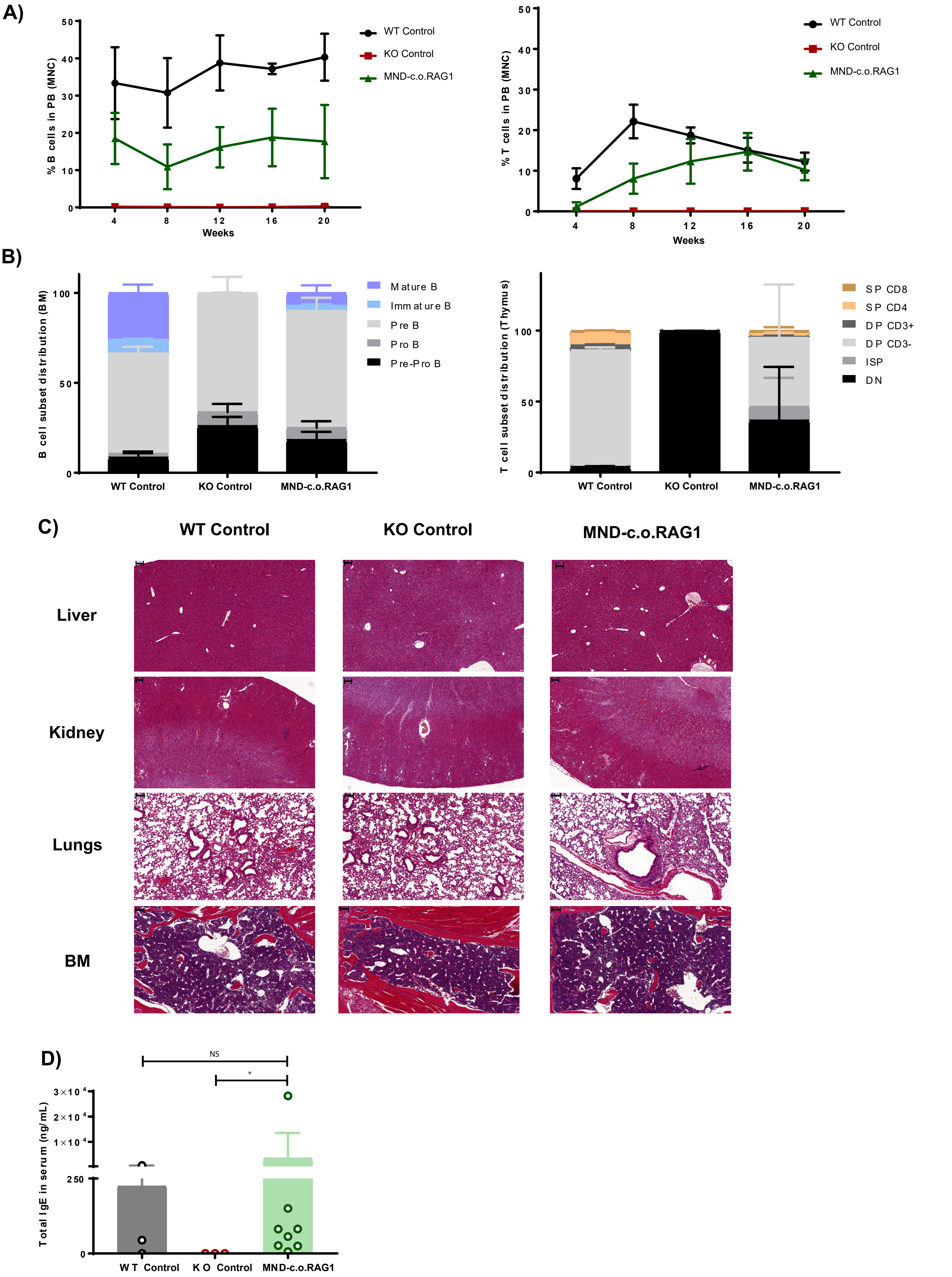

Suppl. Figure 3 Human immune reconstitution after CD34+ MND-c.o.RAG1 transplantation

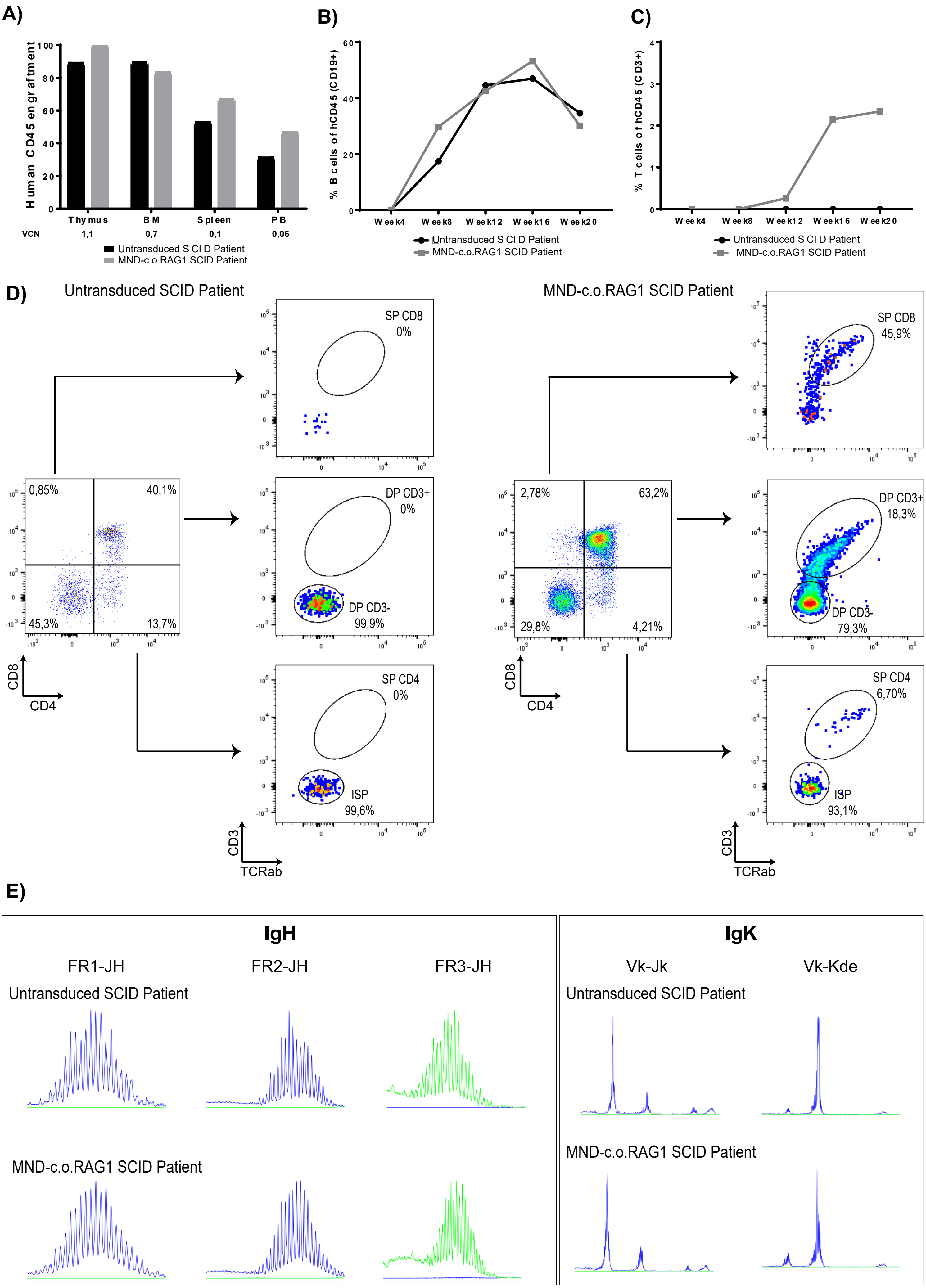

Supplemental Figure:

Figure S1: Choice of the optimal SIN Lentiviral plasmid

A) Percentage of B cells (CD11b/CD43$^-$B220$^+$ cells; left panel) and T cells (CD3$^+$TCRαβ$^+$ cells; right panel) over time in PB after stem cell transplantation with the different constructs (Cbx3.MND-c.o.RAG1, MND-c.o.RAG1, PGK-c.o.RAG1 and UCOE-c.o.RAG1) (Data from 2 independent experiments, total of 6 or 7 mice/group). **B)** Summary of the *in vitro* decision criteria (from 3 independent lentiviral batches) taken into account for the choice of the most optimal plasmid to correct RAG1 deficiency. **C)** Summary of the *in vivo* decision criteria (2 independent experiments with total of 6 or 7 mice per group) taken into account for the choice of the most optimal plasmid to correct RAG1 deficiency.

Figure S2: Immune development after MND-c.o.RAG1 gene therapy in Rag1$^{-/-}$ mouse model

A) Percentage of B cells (CD11b/CD43$^-$B220$^+$ cells; left panel) and T cells (CD3$^+$TCRαβ$^+$ cells; right panel) over time in PB after stem cell transplantation with the clinical MND-c.o.RAG1 batch (3 WT control mice, 3 KO control mice and 8 MND-c.o.RAG1 mice). **B)** B-cell development subsets distribution in BM (left panel) and T-cell development populations distribution in the thymus (right panel) 24 weeks after SC transplantation. Graphs represent the means and standard deviation of 3 mice for control groups and 8 mice in the gene therapy group. **C)** Histologic analysis of the liver (scale bar = 100µm), kidney (scale bar=200µm), lungs (scale bar=100µm) and BM (scale=100µm) stained with hematoxylin and eosin. Representative images from WT Control, KO Control and MND-c.o.RAG1 mice. **D)** Quantification of total IgE in serum by ELISA. Each dot represents a value obtained in one mouse (3 mice/control group, 8 MND-c.o.RAG1 mice). Mann-Whitney test (two tailed, *$p<0,05$; **$p<0,01$)

Figure S3: Human immune reconstitution after CD34+ MND-c.o.RAG1 transplantation

A) Percentage of human chimerism (hCD45$^+$/(hCD45$^+$mCD45$^+$) in immune organs of NSG mice transplanted with CD34$^+$ SCID patient cells and CD34$^+$ SCID patient cells transduced with MND-c.o.RAG1, 24 weeks after transplantation (1 NSG mouse per condition). **B)** Human B-cell percentage (CD19$^+$ cells per total hCD45$^+$ cells) over time in peripheral blood during transplantation. **C)** Human T-cell development (CD3$^+$ cells per total hCD45$^+$ cells) over time in PB during transplantation. **D)** Flow cytometry analysis of thymocytes 24 weeks after transplantation showing T-cell development through the different stages. **E)** Human IgH and IgK repertoire analysis of isolated DNA from NSG BM (SCID patient and SCID MND-c.o.RAG1) using IgH + IgK B-Cell Clonality Assay. (x-axis indicates fragment sizes; y-axis shows the fluorescence intensity of the runoff products).

Table S1
Organs list

Organs	Immune phenotyping	Pathology	Vector Biodistribution
Adrenal gland		x	
Brain		x	x
Cecum		x	
Colon		x	
Duodenum		x	
Gastrocnemius		x	x
Gonalds		x	x
Head-Eyes		x	
Heart		x	x
Ileum		x	
Jejunum		x	x
Kidney		x	x
Limb (front and back)	x	x	x
Liver		x	x
Lung		x	x
Lymph Node (iliac)			x
Lymph Node (Lumbar)		x	
Lymph Node (mesenteric)		x	
Lymph Node (sacral)		x	
Lymph Node (submand.)		x	
Pancreas		x	x
Rectum		x	
Skin		x	
Spinal Cord		x	
Spleen	x	x	x
Sternum		x	
Stomach		x	x
Thymus	x	x	x
Urinary Bladder		x	x

Table S2: Antibody list

Anti-mouse Antibody	Fluorochrome	Clone	Company	Identifier	
				Catalog number	RRID
CD3e	Biotin	145-2C11	BD Bioscience	553060	AB_394593
CD4	PE-Cy7	RM4-5	eBioscience	25-0042-82	AB_469578
CD8a	PerCP	53-6.7	BioLegend	100732	AB_893423
CD11b	Biotin	M1/70	Biolegend	101204	AB_312787
CD19	APC	1D3	BD Bioscience	550992	AB_398483
CD23	Pe-Cy7	B3B4	eBioscience	25-0232-81	AB_469603
CD43	Biotin	S7	BD Bioscience	553269	AB_2255226
CD43	PE	S7	BD Bioscience	553271	AB_394748
CD44	APC-Cy7	IM7	BD Bioscience	560568	AB_1727481
CD45	FITC	30-F11	BD Bioscience	553079	AB_394609
CD45R/B220	PerCP	RA3-6B2	Biolegend	103233	AB_893355
CD45R/B220	Pe-Cy7	RA3-6B2	eBioscience	25-0452	AB_2341160
CD62L	APC	MEL-14	Biolegend	104411	AB_313098
CD93	APC	AA4.1	eBioscience	17-5892	AB_469466
CD138	PE	281-2	BD Bioscience	553714	AB_395000
IgD	ef450	11-26c	eBioscience	48-5993-80	AB_1272239
IgM	FITC	II/41	BD Bioscience	553437	AB_394857
TCRβ	FITC	H57-597	BD Bioscience	553171	AB_394683
TCRγδ	PE	GL3	BD Bioscience	553178	AB_394689
Streptavidin	APC-Cy7	-	BD Bioscience	554063	AB_10054651
Streptavidin	ef450	-	eBioscience	48-4317-82	AB_10359737

Anti-human Antibody	Fluorochrome	Clone	Company	Identifier	
				Catalog number	RRID
CD1a	APC	HI149	BD Bioscience	559775	AB_398669
CD3	FITC	SK7	BD Bioscience	340542	AB_400051
CD3	BV786	SK7	BD Bioscience	563800	AB_2744384
CD4	PE	SK3	BD Bioscience	345769	AB_2728699
CD4	APC-Cy7	RPA-T4	BD Bioscience	557871	AB_396913
CD5	FITC	UCHT2	BD Bioscience	561896	AB_10894588
CD7	Pe-Cy5	M-T701	BD Bioscience	555362	AB_395765
CD8	Pe-Cy7	SK1	BD Bioscience	335805	AB_399980
CD8	BV650	RPA-T8	Biolegend	301041	AB_11125174
CD10	Biotin	SN5c	eBioscience	13-0108-82	AB_763433
CD13	APC	WM15	BD Bioscience	557454	AB_398624
CD16	PE	B73.1	BD Bioscience	347617	AB_400331
CD19	Pe-Cy7	HIB19	eBioscience	25-0199-41	AB_1582279
CD19	APC-Cy7	HIB19	BD Bioscience	557791	AB_396873
CD20	PE	L27	BD Bioscience	346581	AB_400251
CD33	APC	P67.6	BD Bioscience	551378	AB_398502
CD34	PE	8G12	BD Bioscience	348057	AB_400371
CD34	PE-CF594	581	BD Bioscience	562383	AB_11154586
CD34	APC	581	BD Bioscience	555824	AB_398614
CD38	Pe-Cy7	HIT2	eBioscience	25-0389-42	AB_1724057
CD45	V450	HI30	BD Bioscience	560367	AB_1645573

CD45RA	FITC	L48	BD Bioscience	347723	AB_400343
CD45RA	BV510	HI100	BD Bioscience	563031	AB_2722499
CD49f	PerCP-ef710	ebioGoH3	eBioscience	46-0495-82	AB_10670075
CD56	PE	MY31	BD Bioscience	340685	AB_400088
CD56	APC-Cy7	HCD56	Biolegend	318332	AB_10896424
CD62L	BV605	DREG-56	Biolegend	304833	AB_2562129
CD90	APC	5E10	eBioscience	17-0909-42	AB_11042579
IgD	Biotin	IA6-2	BD Bioscience	555777	AB_396112
IgM	PerCP-Cy5.5	MHM-88	Biolegend	314512	AB_2076098
TCRαβ	PerCP-Cy5.5	IP26	Biolegend	306723	AB_2563001
TCRγδ	APC	B1	BD Bioscience	555718	AB_398611
Streptavidin	PerCP-Cy5.5	-	BD Bioscience	551419	
Streptavidin	Pe-Cy7	-	BD Bioscience	557598	AB_10049577

Tissue staining Antibody	Fluorochrome	Clone	Company	Identifier	
				Catalog number	RRID
FOXP3 Antibody , ABfinity™ Rabbit Monoclonal	-	5H10L18	Thermofisher	700914	-
Keratine 5/6 Monoclonal	-	D5/16 B4	Dako	GA780	-

ELISA Antibody	Fluorochrome	Clone	Company	Identifier	
				Catalog number	RRID
Anti-mouse IgG	Unlabeled	-	SouthernBiotech	Cat# 1030-01	-
Anti-mouse IgM	Unlabeled	-	SouthernBiotech	Cat# 1020-01	-
Anti-mouse IgE	Unlabeled	-	SouthernBiotech	Cat# 1110-01	-
Anti-mouse IgG	Biotin-conjugated	-	SouthernBiotech	Cat# 1030-08	-
Anti-mouse IgM	Biotin-conjugated	-	SouthernBiotech	Cat# 0101-08	-
Anti-mouse IgE	Biotin-conjugated	-	SouthernBiotech	Cat# 1002-08	-
Anti-human IgM	Unlabeled	-	Jackson Immuno Research Laboratories	Cat# 109-005-129	-
Anti-human IgM	Biotin-conjugated	-	Novex Life Technologies	Cat# A24486	-
Streptavidin HRP		-	Jackson Immuno Research Laboratories	Cat# 016-030-084	-

Table S3
List of primers and probes

Determination VCN and c.o.Rag1 expression

Description	Orientation	DNA sequence 5'-3'
ABL1	FW	5’-TGGAGATAACACTCTAAGCATAACTAAAGGT-3’
	RV	5’-GATGTAGTTGCTTGGGACCCA-3’
	Probe	5’FAM-CCATTTTTGGTTTGGGCTTCACACCATT- TAMRA 3’
c.o.Rag1	FW	5' CAACTGCAAGCACGTGTTCTG 3'
	RV	5' GCAGTAGCTGCCCATCACTTT 3'
	Probe	5'FAM AGAGTGTGCATCCTGCGGTGCCT TAMRA 3'
PTBP2	FW	5’-TCTCCATTCCCTATGTTCATGC-3’
	RV	5’-GTTCCCGCAGAATGGTGAGGTG-3’
	Probe	[JOE]-ATGTTCCTCGGACCAACTTG-[BHQ1]
WPRE	FW	5’- GAGGAGTTGTGGCCCGTTGT-3’
	RV	5’-TGACAGGTGGTGGCAATGCC-3’
	Probe	[6FAM]-CTGTGTTTGCTGACGCAAC-[BHQ1]

Repertoire Analysis (murine)

Description	Orientation	DNA sequence
***V* gene segment-specific oligonucleotide**		**(5’ -> 3’, coding strand)**
mVβ1	FW	CTGAATGCCCAGACAGCTCCAAGC
mVβ2	FW	TCACTGATACGGAGCTGAGGC
mVβ3.1	FW	CCTTGCAGCCTAGAAATTCAGT
mVβ4	FW	GCCTCAAGTCGCTTCCAACCTC
mVβ5.1	FW	CATTATGATAAAATGGAGAGAGAT
mVβ5.2	FW	AAGGTGGAGAGAGACAAAGGATTC
mVβ5.3#	FW	AGAAAGGAAACCTGCCTGGTT
mVβ6	FW	CTCTCACTGTGACATCTGCCC
mVβ7	FW	TACAGGGTCTCACGGAAGAAGC
mVβ8.1	FW	CATTACTCATATGTCGCTGAC
mVβ8.2	FW	CATTATTCATATGGTGCTGGC
mVβ8.3	FW	TGCTGGCAACCTTCGAATAGGA
mVβ9	FW	TCTCTCTACATTGGCTCTGCAGGC
mVβ10	FW	ATCAAGTCTGTAGAGCCGGAGGA
mVβ11	FW	GCACTCAACTCTGAAGATCCAGAGC
mVβ12	FW	GATGGTGGGGCTTTCAAGGATC
mVβ13	FW	AGGCCTAAAGGAACTAACTCCCAC
mVβ14	FW	ACGACCAATTCATCCTAAGCAC
mVβ15	FW	CCCATCAGTCATCCCAACTTATCC
mVβ16	FW	CACTCTGAAAATCCAACCCAC
mVβ17#	FW	AGTGTTCCTCGAACTCACAG
mVβ18	FW	CAGCCGGCCAAACCTAACATTCTC
mVβ19#	FW	CTGCTAAGAAACCATGTACCA
mVβ20	FW	TCTGCAGCCTGGGAATCAGAA
**C gene segment specific oligonucleotide**		**(5’ -> 3’, non-coding strand)**
muTCB1-FAM	RV	FAM-CTTGGGTGGAGTCACATTTCTC
